# Knowledge mobilisation: a UK co-creation study to devise strategies to amend lay and practitioner atopic eczema mindlines to improve consultation experiences and self-management practices in primary care

**DOI:** 10.1136/bmjopen-2019-036520

**Published:** 2020-09-28

**Authors:** Fiona Cowdell, Taheeya Ahmed, Carron Layfield

**Affiliations:** 1 Faculty of Health, Education and Life Sciences, Birmingham City University, Birmingham, UK; 2 Centre of Evidence Based Dermatology, University of Nottingham, Nottingham, UK

**Keywords:** eczema, dermatology, primary care, qualitative research

## Abstract

**Objective:**

To devise strategies to amend lay and practitioner atopic eczema mindlines ‘collectively reinforced, internalised tacit guidelines’, to improve consultation experiences and self-management practices in primary care.

**Design:**

Co-creation workshops informed by the Co:Create Coproduction Matrix.

**Setting:**

Conference centre in central England and via remote communication.

**Participants:**

Lay people with, and parents of children with, atopic eczema, practitioners, a researcher and a facilitator (n=22).

**Results:**

Eczema mindline amendment needs to address people and parents of children with the condition, practitioners and wider society in parallel. For lay people trust and *‘*realness’ of amendment activity was vital and practitioners wanted practical, locally relevant, hints and tips, tailored, ‘no faff’ approaches. To improve consultation experiences and self-management practices, five key, consistent, evidence-based messages need to be instilled into eczema mindlines: (1) eczema is more than just dry skin, (2) eczema does not just go away, (3) moisturisers are for every day, (4) steroid creams are okay when you need them and (5) you know your child’s eczema best.

**Conclusion:**

This co-creation study provides original insights into *what* eczema knowledge should be mobilised, *who* needs to have this knowledge, *how* this should be achieved to amend existing mindlines to improve consultation experiences and self-management practices in primary care.

The remaining challenge is to refine, implement and evaluate the effectiveness of strategies developed to instil the five core messages and erase outdated or inaccurate information.

**Strengths and limitations of this study**

- First co-creation study to examine strategies to amend lay–practitioner social atopic eczema mindlines.
- Diverse lay and practitioner co-creation group.
- Only those with an existing interest in eczema joined the co-creation group.

## Introduction

Atopic eczema (hereafter eczema) is a common burdensome long-term skin condition^1^ with a high self-management demand.^2^ Evidence-based treatment guidance is available, for example, from the National Institute for Health and Clinical Excellence^3^ and Clinical Knowledge Summaries.^4^ The mainstay of treatment is use of topical corticosteroids (TCS) at times of flare and regular, consistent application of emollients even when the skin appears healthy; in essence ‘getting control and keeping control’.^5^ Eczema management is 97% primary care based in the UK.^6^ Primary care consultations are often unsatisfactory for patients and practitioners alike.^7 8^ Patients report practitioners with limited knowledge^9^ and dismissive attitudes.^10^ Some practitioners describe uncertainity about optimum treatment^11^; others regard eczema as simple to treat and perceive, no need to change their current treatment ‘recipe’.^12^ A particular challenge is ensuring safe and appropriate use of TCS. Steroid phobia is common^13^ and reinforced at many levels^12 14^ potentially leading to under treatment, unwarranted suffering or treatment escalation^15^ and wastage of prescribed medication.^16^ Eczema is a long-term condition, often with a high self-management demand. Long-term condition self-management is a policy imperative.^17–19^ Definitions of self-management vary^20^ but involve ongoing efforts to maintain or improve health. Self-management interventions are designed to increase a person’s capacity, confidence and efficacy of to perform the necessary activities.^21^ Self-management of long-term skin conditions is notoriously challenging.^22^ Supportive interventions include bespoke programmes^23^ and educational and psychological interventions.^24–26^ However, such offerings are not available to all, are costly and impact is inconsistent.^23^

Knowledge mobilisation (KM) is essentially ‘moving knowledge to where it can be most useful’^27^; it involves deliberative actions to create, disseminate and operationalise research and other forms of knowledge.^28^ KM is context specific,^29^ relational^30^ and socially constructed.^31^ One approach to mobilising knowledge is through amending mindlines.^12 14 32^ Mindlines are ‘collectively reinforced, internalised tacit guidelines’, which underpin clinical decision-making.^33^ They are built on a multifaceted combination of knowledge sources such as communication with colleagues and opinion leaders in the field and from personal tactic knowledge developed over time.^33^ Mindlines are founded in the work of Polyani^34^ and Nonaka and Takeuchi^35^; these authors recognise that knowledge is not necessarily conscious and explicit, but is in large part tacit and created from technical know-how and unconscious schemata. In practice, this tacit knowledge is a far more influential than formal codified knowledge.

The seminal mindlines paper of Gabbay and le May^36^ has been cited 945 times to date. An extensive review a decade later^37^ reveals that little attention has been paid to condition specific mindlines or to a patient equivalent to mindlines, although Gabbay and le May^33^ intimate their existence that they do not develop this notion. Repeating the review search strategy reveals a continued absence of focus on patient mindlines with the exception of recent ethnographic work by one of the authors of this paper (FC), elucidating lay and practitioner eczema mindlines.^12 14 32^ These studies advocate improving eczema consultation experiences and self-management practices in primary care through a concerted effort amend eczema mindlines to increase shared knowledge, understanding and decision-making. It is proposed that this may be achieved through parallel lay and practitioner mindline amendment.

**Figure 1:**
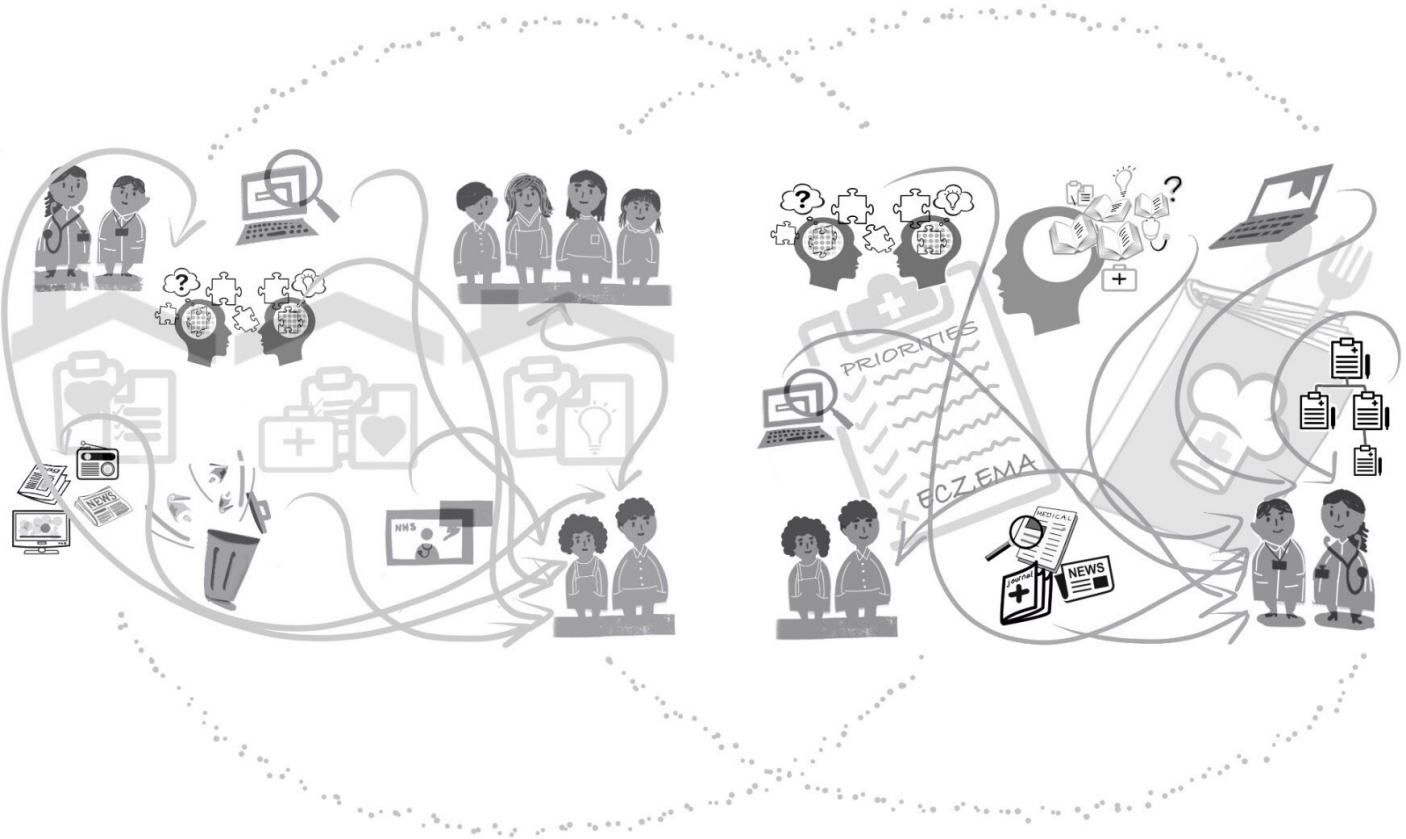
Lay (left side) and practitioner (right side) eczema mindlines and inter-relationship between the two.

Mindline amendment requires collaborative efforts from lay people, that is, people with and parents of children with eczema, practitioners and researchers. Figure 1 illustrates the fundamentals of eczema mindlines and shows the complexity of how knowledge is gained, from which sources and the inter-relationship between lay–practitioner wider society mindlines. In essence, (1) lay people gain knowledge and beliefs from many sources, for example, personal experience, family, friends and wider society, trial and error, online and from practitioners^14^; and (2) practitioner actions are underpinned by a belief that eczema is simple to treat *‘*the recipe does not change*’* and that treatment options are limited by prescribing guidance, knowledge is accrued from early education, colleagues, practical experience and patients.^12^ Further work illuminates the inter-relationship between the two and therefore the need for efforts to amend mindlines to be a shared venture which transcends lay–practitioner boundaries and instils shared and consistent understanding.^32^

Co-methodologies in healthcare are increasingly considered to be a ‘good thing’. The language of ‘coworking’ remains contested.^38 39^ There is a multitude of models but it is increasingly considered that coworking should involve lay people and professionals working as equals at every stage of the research process.^39 40^ Essential questions when planning co-creation include ‘who is participating, in what and for whose benefit’.^41^

In light of the prevalence of eczema, the high self-management demand, the challenges of primary care consultations and recent developments in understandings of lay and practitioners eczema mindlines,^12 14 32^ it seems prudent to investigate the way in which co-creation may be used to devise novel approaches to influence these mindlines. The intention is to mobilise relevant, accurate, up to date and contextually appropriate knowledge to support positive primary care consultations and enable people with eczema to self-manage as effectively as possible.

## Methods

### Aim

To devise strategies to amend lay and practitioner eczema mindlines to improve consultation experiences and self-management practices in primary care.

### Objectives

1. To agree what a good consultation and effective self-management looks like.
2. To identify
  1. *What* knowledge needs to be mobilised.
  2. *Who* needs this knowledge.
  3. *How* should this knowledge be shared.

### Design

We employed a co-creation approach informed by the ‘Gold Standard’ Coproduction Matrix.^40^ which comprises eight principles as summarised in box 1.

Box 1 **Eight principles of the Co:Create Coproduction Matrix**

- Holistic: Coproduction should happen at every stage.
- Resourced: Meaningful and effective coproduction deserves and requires sufficient resource.
- Transparent: Coproduction should have a clear and transparent remit that is,: overall aims, limitations, expectations and commitment.
- Inclusive: Coproduction should involve a wide range of people (eg, practitioners, customers, future users, the wider community), capturing individual and differing views.
- Iterative: Coproduction should be reciprocal, repeated and progressive, always adapting and building uponon what came before.
- Positive: Coproduction should be mutually beneficial and an overall positive experience.
- Equal: Each participant and their contribution should be valued equally.
- Sustainable: Meaningful coproduction should have a genuine sustainable impact on the project.

### Setting, co-creators and process

The co-creation group was recruited via a central England Higher Education Institute website with mass sharing via word of mouth and media posts, professional networks and an existing dermatology patient panel. Maximum variation purposive sampling^42^ was applied in selecting co-creators of different age, gender and ethnicity, with experience of giving or receiving eczema care, ensuring a balance of lay and practitioner participants (n=22). Potential co-creators were sent an information sheet. Those who agreed to take part received a copy of the original mindline publication of Gabbay and le May^36^ and a copy of the lay and practitioner eczema mindline illustrations together with a brief explanation of how these had been developed and confirmed. The group met for a series of three face-to-face workshops and iterative email exchanges between April 2018 and March 2019. Workshops took place outside usual working hours at an accessible conference centre. The researcher (FC) and facilitator (CL), both of whom are experienced in co-creation, attended all three workshops. Practitioners attended workshop one, lay people with eczema, or who had children with eczema, attended workshop two and the full group attended workshop three. Following each workshop, data were summarised and emailed to group members for discussion and approval. After the third workshop, final refinement of ideas and terminology for key messages was achieved via email.

Although co-creators were nominally either lay or professional there was a degree of overlap, for example, two of the practitioners had children with eczema and one lay person was also a practitioner. Demographic details are summarised in table 1.

**Table 1:** Demographic details of the co-creation group

Role	Gender	Age range
Lay person	F	35–44
Lay person	M	Under 16
Lay person	F	Under 16
Lay person	M	Over 55
Lay person	F	Over 55
Lay person	F	25–34
Lay person	F	45–54
Lay person	F	25–34
Lay person	F	45–54
Lay person	F	35–44
Dermatology specialist nurse	F	45–54
General practitioner	F	25–34
General practitioner trainee	M	25–34
Practice nurse	F	45–54
Medical student	F	17–24
Pharmacist	M	25–34
General practitioner trainee	F	25–34
Primary care nurse	F	45–54
Pharmacist	M	45–54
Pharmacy counter assistant	F	35–44
Facilitator	F	45–54
Researcher, facilitator and nurse	F	45–54

### Data collection

Data collected included: facilitator notes written throughout the session, summaries of individual and group presentation information recorded on flip chart, checked by group members at the time of writing and artefacts, for example, group member notes and products from activities. Details of workshops are summarised in table 2.

**Table 2:** Details of co-creation workshops

Workshop	Attendees	Time	Activity
1	Practitioners, researcher and facilitator	2.5 hours	Introductions and clarification of purpose of workshop. Discussion and questions about the circulated article and eczema mindlines illustrations. Selection from a choice of ordinary postcards that co-creators thought represented a ‘good’ eczema consultation; each person briefly presented their thoughts. Small group exercise to identify behaviours that patients and practitioners should start, stop and continue to bring about an improvement in eczema consultations and self-management. Identification of initial priorities for mindline amendment.
2	Lay people, researcher and facilitator	3 hours	As above.
3	Lay people, practitioners, researcher and facilitator	1 day	Introductions and clarification of purpose of workshop. Recap and discussion about the process and outcomes of workshops one and two. Co-creators individually reviewed priorities for mindline amendment derived from sessions 1 and 2 (table 3) and ranked these in order of which they considered most likely to lead to improvement in consultation experience and self-management. Individuals worked in three mixed subgroups to identify their top three priorities. Whole group reconvened and each subgroup presented their rationale and choice for their top three after which, through consensus activity, a final three priorities for action were agreed. Subgroups worked with a range of creative resources to contemplate how lay and practitioner eczema mindlines may best be amended.

**Table 3:** Priorities for and facets of eczema mindline amendment

Priorities for mindline amendment	Facets
Prioritise eczema	Be attentive, make eczema the primary reason for consultation, offer or go for follow-up.
Manage eczema as a long-term condition	Educate, explain, avoid quick fixes, do not expect a cure, ‘get control, keep control’.
Prepare for each consultation	Be aware of patient history, offer facts and good explanation, plan what to say, know what you want to achieve.
Be consistent with treatment	Be concordant, know products and how to use them, agree realistic regimens, be truthful, do not waste, try over the counter products, understand local formulary.
Work together	Listen and question, acknowledge expertise, understand burden of treatment, plan, get control, keep control.
Get the right emollient	Be familiar with products, offer choice, agree feasible regimen.
Use topical steroids appropriately	Understand risk and benefits, use for flares, use the best product for the optimum time.

### Data analysis

Data collection and analysis were iterative processes.^43^ The researcher (FC) transcribed data from workshops and reviewed this as whole prior to summarising. FC and CL analysed the data summaries identifying key points and potential areas for mindline amendment as generated by the group. We paid attention to areas of agreement and disagreement between individuals and groups and circulated data summaries to group members for comment, supplementation and modification.

### Reflexivity

A reflexive stance was maintained for the duration of the study with particular consideration being given to subjectivity and positioning of the researcher as a nurse with a particular interest in skin health; pre-understandings were consciously set aside.^44^

### Patient and public involvement

Lay people were involved in the development of the research question, planning and delivering the study and disseminating results.

## Results

The co-creation group addressed four question. Each is discussed below with explanatory examples from the data.

1. What makes a good eczema consultation in primary care?

Lay group members suggested that a good consultation for them required a practitioner who understood the impact of eczema on their lives and had time to discuss these issues, ‘usually not enough time to talk about the condition, how it effects life, more talking and understanding would be a good consultation’. Honesty about their knowledge was valued even when limited, provided they ‘point us to useful information, the right direction and resources’. In parallel was the need for practitioners to respect individual’s expertise in their own condition, ‘I have an understanding, long-term and understand when I need help and something different’. Group members wanted to see the same practitioner when possible to avoid feeling ‘sick and tired of saying the same story over and over again’. They wanted practitioners to take eczema seriously, think of it as a long-term condition, ‘to help manage ‘long-term’ rather than short-terms solution for flares’. Group members accepted their role in self-management with even the youngest stating the need for ‘taking responsibility and sharing responsibility for condition……… makes you grow up’. While embracing their contribution to long-term eczema control group members wanted to work with practitioners and to ‘come to an understanding, work together, look at self-management…but…be there for support if needed’. They desired a sense of ‘we can manage this, do not want to feel alone’.

For practitioners, a good eczema consultation was characterised by empathy, with two-way dialogue between ‘equals’ to generate shared understanding of eczema and treatment history asking *‘*tactful question*s*’ so that both parties understand and ‘play the same tune, be on the same page’. Consistency in message from practitioners reduced misunderstandings and enhanced patient’s confidence. They spoke of the long-term nature of eczema and the need to be ‘blunt (honest!)’ about the need to ‘explain long-term use of emollients to avoid flares’. Agreeing goals to ‘get control and keep control’ was pivotal, with some advocating the provision of written information including action plans. Strategies to ‘work with patients and motivate them to use emollient*s*’ and ‘emphasising long-term gain’ were promoted. It was important to find the ‘right’ emollient. Being flexible to patient preference and experience was vital, although difficult within the confines of local prescribing guidelines.

2. What should lay people and practitioners start, stop and continue to improve eczema consultations and self-management?

Co-creators identified the aspects of consultations that should be started, stopped and continued from both perspectives. Common threads included the need for a long-term approach to care, being prepared for the consultation, prioritising eczema, shared decision-making and consistent use of topical treatments (see online supplemental information 1 for more detail). Of note was that the group agreed that general practitioners (GPs) were not necessarily the right practitioner to manage eczema and that more attention needs to be given to the role of other professionals, specifically community pharmacists, pharmacy counter assistants, nurses and health visitors.

10.1136/bmjopen-2019-036520.supp1 Supplementary data

3. What are the priority areas for lay and practitioner eczema mindline amendment?

Distillation of data collected from the first two workshops and associated email exchanges identified seven key areas for mindline amendment: (1) prioritise eczema, (2) manage eczema as a long-term condition, (3) prepare for each consultation, (4) be consistent with treatment, (5) work together, (6) get the right emollient and (7) use steroids appropriately. The facets of each area for mindline amendment are summarised in table 3. Each area was viewed from both lay and practitioner perspectives.

Following a consensus activity, the group prioritised three areas for mindline amendment: (1) manage eczema as a long-term condition, (2) work together and (3) use topical steroids appropriately.

4. How may lay and practitioner eczema mindlines best be amended?

The group highlighted challenges in modifying mindlines. These included the belief that eczema is not a priority for practitioners, some beliefs are deeply entrenched, and some reluctance to engage in shared decision-making. In essence amendment activities suggested were multifaceted. As each idea was proposed, pros and cons were identified and many discounted as not feasible for reasons such as lack of resources, for example, time and funding.

All agreed that interventions to amend the mindlines of either group in isolation would not be effective as they are inextricably linked. Co-creators identified the influence of wider social influences on beliefs about eczema care, for example, in promulgating powerful messages, such as children ‘grow out of eczema’ and the myth that topical steroid preparations are necessarily ‘dangerous’. For this reason, they suggested that any attempt to amend mindlines would have to address all key players in parallel. For lay people trust and ‘realness’ of amendment, activity was vital and practitioners wanted practical, locally relevant, hints and tips, tailored, ‘no faff’ approaches.

While amendment of knowledge was deemed important, the group was unanimous in agreeing that providing information alone is not sufficient. There is also a need to redress power imbalances between patient and practitioner and promote shared understanding and decision-making. With this in mind, email exchanges with co-creators resulted in the three priorities being translated into five key, consistent, evidence-based messages: (1) eczema is more than just dry skin, (2) eczema does not just go away, (3) moisturisers are for every day, (4) steroid creams are okay when you need them and (5) you know your child’s eczema best. The latter was added as it was considered that initial mindline amendment activity should be targeted and, given the prevalence of childhood eczema, this was a reasonable focus. It also acknowledges the importance of parents’ knowledge and expertise. Each priority is underpinned by a set of simple, illustrated messages (online supplemental information 2). Given the need for consistency, clarity and straightforwardness of messages, at this stage we revisited existing evidence-based resources including National Institute for Health and Care Excellence (NICE) guidance,^3^ clinical knowledge summaries (CKS)^4^ and also consulted with the National Eczema Society to ensure consistency and fit with existing evidence base.

10.1136/bmjopen-2019-036520.supp2 Supplementary data

Co-creators worked with a range of resources to develop potential methods of amending lay and practitioner eczema mindlines; initial ideas for further development are summarised in box 2.

Box 2 **Initial ideas for mindline amendment strategies**

- Holding ‘pop ups’ of eczema information in places not currently targeted.
- Having simple information available in skin care sections of supermarkets.
- Targeting information at ‘Health Living Pharmacies’.
- Using ‘Steroid Sam the emoji’ in information to illustrate topical steroids in a more positive light.
- Making more use of the Patient Oriented Eczema Measure/Children’s Dermatology Life Quality Index so parents can be more confident in ‘proving’ impact of eczema during consultations.
- Offering a ‘recipe’ for better skin.
- Build on existing resources, for example, Dr Ranj’s work.

https://www.youtube.com/watch?v=lkIekfkoSbI

https://www.youtube.com/watch?v=xM6XaL0gaho

## Discussion

The aim of this co-creation study was to devise strategies to amend lay and practitioner eczema mindlines to improve consultation experiences and self-management practices in primary care. Amending mindlines offers an entirely new approach to changing eczema care, which goes beyond existing education interventions for patients and parents.^23–26^ It also addresses the identified challenges of primary care practitioners not prioritising their own eczema education.^12^ In co-creation, particular emphasis was placed on defining *what* knowledge needs to be mobilised, w*ho* needs this knowledge and *how* should this knowledge be shared. These questions were answered through a series of structured co-creation workshops and virtual communications.

*What* knowledge needs to be mobilised? Although most lay people and practitioners know the fundamental ‘ingredients’ of eczema care are TCS and emollients, it is knowledge about the nuanced ‘recipe’ for most effective use that needs to be amended. Alongside this, the long-term nature of eczema and the need for mutual understanding between lay person and practitioner must be understood. *Who* needs this knowledge? Mindline amendment activity needs to be comprehensive, engaging people and parents of children with the condition, the full gamut of practitioners and people in wider society who may influence eczema care. *How* should this knowledge be shared? Importantly, knowledge alone is not sufficient to improve consultation experiences and self-management practices. There is a parallel need to change the balance of power in patient–practitioner relationships. These results are reflected in the five key messages to underpin eczema mindline amendment (see above).

This original study is one of the first to investigate how condition specific mindlines may best be amended across lay–practitioner social boundaries. A robust approach to co-creation is demonstrated thorough adherence to the Gold Standards for co-creation.^40^ In particular, the process was well resourced, the group was diverse in terms of age, gender, ethnicity and expertise and was able to agree mutual aims and objectives and consistently and equally work together to achieve these. The co-creative process was reciprocal and progressive, steadily building on previous work to develop a sustainable approach to eczema mindline amendment. Reporting is in accordance with the consolidated criteria for reporting qualitative research.^45^

Limitations are twofold, first only those with an interest in eczema were likely to take part, thus neglecting the many people who do not prioritise eczema care. Second, face-to-face co-creative time was limited, but this was mitigated by skilled facilitation, careful planning to ensure best use was made of available time and by using follow-up emails and conversations for clarification and development.

The need to address knowledge deficits, embrace key influencers across lay–practitioner social boundaries and power imbalances led to the development of five key messages together with potential modes of delivery intended to bring about widespread changes in eczema mindlines. These are discussed in relation to the existing literature. Findings from this present study reflect existing research that reports poor consultation experiences, practitioners with limited knowledge and apparent apathy and lack of priority given to eczema management by both some lay people and some practitioners.^9–11^ This mirrors the state of ‘gloom à deux’ in which patients and practitioners share a perception of hopelessness,^46^ which is inimical to effective care. As with Gabbay and le May,^33^ the present study points to the inter-relationship of patient–practitioner mindlines. Our findings are in accordance with and extend the findings of Gabbay and le May^33^ who suggest that in a consultation two sets of mindlines converge as a single instantiation, which is co-constructed during in the encounter. Our study reveals the need to manage this influence directly if eczema care is to improve, a more definitive call than the hint at social influences on healthcare and actions that subsequently occur.^33^

To date, little has been written about mindline amendment strategies; however, there are elements of our co-created approaches that are congruent with the existing literature. The need to provide clear, simple, ‘real’ messages from trusted sources to a lay audience beyond those directly affected by eczema, but who may nevertheless influence use of treatments, can be grounded in the literature of social marketing (SM) in healthcare. SM amalgamates concepts from commercial marketing and social sciences; it goes beyond simply imparting knowledge widely and is intended to directly influence healthcare actions.^47^ SM in healthcare has been used internationally since the mid-1970s^48^ and is considered a potential strategy to improve public health, for example, in reduction of smoking and obesity.^49^ It embraces health communication techniques based on mass media. Messages can be mediated through other sources, in our case potentially practitioners and lay influencers. Communication approaches are varied and may include targeted message placement, promotion, dissemination and community outreach.^50^ While evidence of the effectiveness of SM in healthcare remains sparse,^51^ it is argued that some elements have the potential to support lay mindline amendment, given the high prevalence of childhood eczema, the aim of changing behaviour and the need to get simple, consistent messages to a diverse range of people who have potential to influence treatment use (eg, grandparents, friends and informal contacts).

Approaches to practitioner mindline amendment need to offer ‘no faff’, locally relevant, tailored hints and tips. This need is driven both by the low priority given to eczema and the increasingly heavy workloads of primary care practitioners.^52–55^ Previous studies identify what is important to practitioners if messages are to influence their practice. Utility of information is measured by some in terms of relevance x validity÷work required to obtain it.^56^ Evidence suggests that to influence practice, knowledge must (1) relate to the day-to-day challenge of having to manage this patient, at this time and in this situation,^57^ (2) be accessible, understandable and enable practitioners to make a ‘good enough’ treatment decision and (3) fit with the particularities of the context.^29 58^ Knowledge that addresses these points allows practitioners to transform it through contextual adroitness into knowledge-in-practice-in-context, which enables good clinical care.^59^ Strategies to amend practitioner mindlines that take into account the needs outlined above may include, for example, use of aphorisms, that is, succinct sayings that offer advice and convey concentrated wisdom^60^ or in a similar vein to ‘actionable nuggets’, snippets of practical and memorable information that can be readily used in everyday practice.^61^

Although knowledge is essential, changing this facet of mindlines alone will not change eczema care. Power differentials between lay person and practitioner also need to be minimised. In agreement with the existing literature, our study concludes that knowledge is power^62^ does not hold true in primary care eczema consultations. While practitioners often have access to more clinical information, patients are frequently experts in their own condition.^63^ In common with the existing literature, our group members agreed that when patients are engaged in healthcare decision-making, outcomes^64^ and levels of satisfaction^65^ improve. The core messages for all we designed offer common language^66^ and are intended to reduce ‘informational inequality’^67^ and thus improve shared understanding and agreed, realistic plans of care.

Efforts to influence the mindlines of all types of healthcare provider are intended to support consistency of information. This approach also addresses power imbalances by widening the range of practitioners with whom lay people may choose to consult. Power imbalances are most frequently problematic in consultations with doctors, which begs the question whether they are best placed to provide eczema care. Eczema consultations with expert dermatology nurses are known to be highly valued by patients.^68–71^ However, there are few primary care dermatology nurses in post. There are suggestions that community pharmacists and pharmacy counter assistants, who are often the first point of contact and who can offer advice independent of the pharmacist,^72^ are not yet being fully used.^73–75^

## Conclusion

This study, as with previous research, emphasises the need to improve eczema primary care consultation experiences and self-management practices. As previously noted, approaches to date have predominantly been provision of educational and psychological interventions or the use of written action plans. The present study offers an alternative approach in mindline amendment in which simple, consistent, evidence-based knowledge is shared across patient–practitioner social boundaries to promote shared understanding and which, importantly, acknowledges role of influential members of the wider community. The challenge now is to take the five key messages, underpinned by hints and tips and convert them into a range of formats that can be shared among relevant parties. The impact of this intervention is intended to be revision or modification of mindlines achieved by adding reliable and useful knowledge and erasing outdated or inaccurate information. Further research is needed to assess what bearing it has on primary care eczema consultation experiences and self-management practices.

## Supplementary Material

Reviewer comments

Author's manuscript
