# Supplementary material for: Knowledge mobilisation: a UK co-creation study to devise strategies to amend lay and practitioner atopic eczema mindlines to improve consultation experiences and self-management practices in primary care
Source: BMJ Open. 2020 Sep 28;10(9):e036520. doi: 10.1136/bmjopen-2019-036520 (PMC7523205; doi:10.1136/bmjopen-2019-036520)
Supplement: Supplementary data [file bmjopen-2019-036520supp001.pdf]

BMJ Open Co-creation submitted Dec 2019

**Practitioners suggested that lay people should ………….**		
**Start**	**Continue**	**Stop**
Adhering to regular emollient use	Asking questions and challenging the practitioner	Having eczema as a secondary reason for consultation
Be truthful about their concerns	Going to see their practitioner	Stopping regular emollients
Being forceful if not being listened to, asking questions	Asking for advice	Agreeing to care plans they could or would not follow
Committing to the agreed plan of care	Being invested in their skin, wanting to look after it	Waiting for flares before coming for review
Accessing the evidence not the gossip	Be honest with self and practitioner	Not persevering with advice
Realising it's a chronic condition	Listening, writing it down	Using known "triggers"
Finding the right emollient for them	Owning their bodies	Listening to the ill informed
**Practitioners suggested that practitioners should ………….**		
**Start**	**Continue**	**Stop**
Recognising their limitations, accessing education and referring for help	Educating, explaining, supporting and reinforcing messages	Dismissing it as "only" eczema
Explain *how* and *when* to use treatments, monitor side effects and efficacy and give practical tips on useage	Validating concerns	Prescribing products that are impossible to use
Advising about over-the-counter products	Prescribing sufficient emollient prescribing	Viewing all patients as the same
Listening and empathising	Reiterating it's a long-term condition	Thinking eczema is one issue, it effects more than just skin
Signpost or refer for further information or treatment	Reassuring, there will be flares and good times	Under-prioritising how eczema affects patient's lives
Explaining chronicity, encourage regular review	Provide treatment options	Not looking at patient history relevant to consultations
Be familiar with products and provide patients with a selection of emollients so they find the best for them	Myth busting	Presenting cheapest option that doesn't fulfil patient need
Lobbying for products people can use	Recognising impact of eczema on quality of life	Underusing steroids
**Lay people suggested that lay people should ………….**		
**Start**	**Continue**	**Stop**
Preparing for the consultation, what do I want to achieve?	Providing updates	Assuming they know all the answers
Talking to them about long-term care	Choosing the right practitioner for the problem	Expecting a cure
Asking confidently, robustly for what you need and expecting questions to be answered	Trying what you have been asked to try irrespective of first impressions	Holding back, feel confident to say what needs to be said
**Lay people suggested that practitioners should ………….**		
**Start**	**Continue**	**Stop**

Cowdell F, *et al. BMJ Open* 2020; 10:e036520. doi: 10.1136/bmjopen-2019-036520

Being more approachable to patients knowledge, requests and understanding	Prescribing what works for the patient	Fobbing us off
Explain *how* to apply emollients and corticosteroids, explain the options	Prescribing as the patient wants	Trying to manage it in primary care if you have reached your limit
Being more realistic about the burden of treatment	Educating themselves clinically	Trivialising and dismissing the condition
Referring patients when prescribed treatments are not sufficient	Being open minded, each patient is unique	Telling us not to scratch
Having a better understanding of medication that's available	Educate themselves about the impact on lives	Treating just our skin, treat the whole person
Knowing the person's eczema history	Continue to educate themselves clinically	Thinking about cost so much
Be equipped with patient history	Value the patient's experience	Making false promises

Supplementary information 1: Practices that lay people and practitioners should start, stop and continue to improve consultation experiences.
