# Supplementary material for: Knowledge mobilisation: a UK co-creation study to devise strategies to amend lay and practitioner atopic eczema mindlines to improve consultation experiences and self-management practices in primary care
Source: BMJ Open. 2020 Sep 28;10(9):e036520. doi: 10.1136/bmjopen-2019-036520 (PMC7523205; doi:10.1136/bmjopen-2019-036520)

Eczema is more than just dry skin

- 1 in 5 babies and children have atopic eczema
- A tendency to atopic eczema often runs in families
- There is no one cause
- Faulty skin barrier means the skin is dry, itchy and inflamed
- Eczema is made worse by 'triggers' which can be difficult to avoid

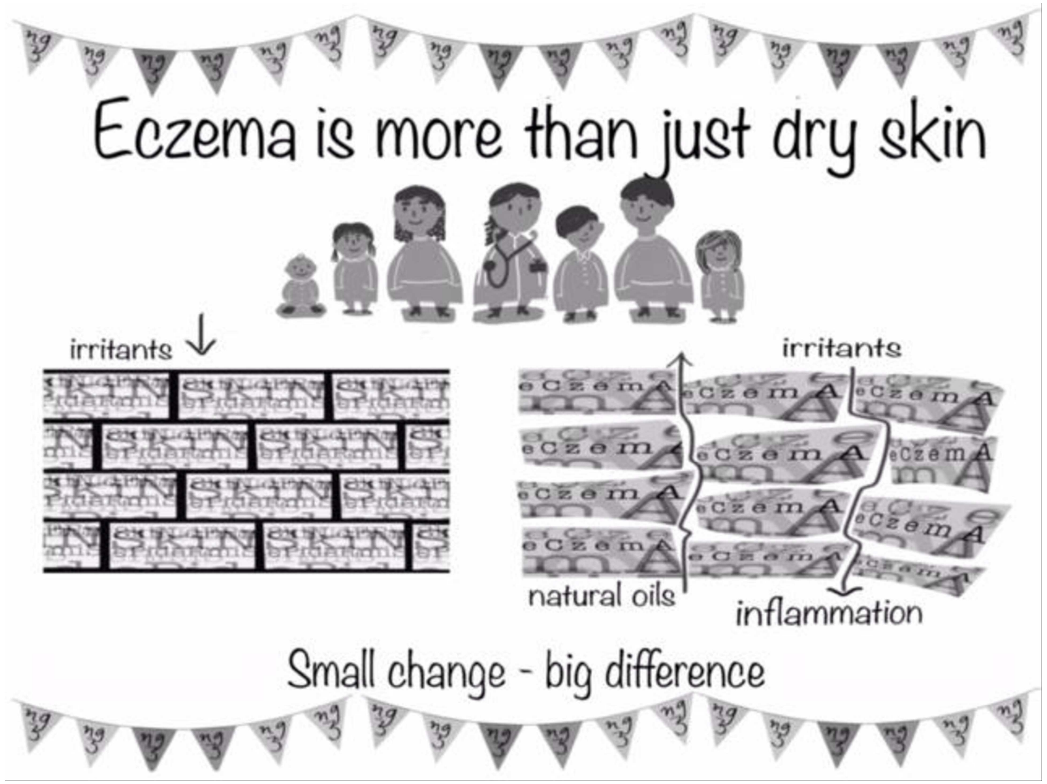

Eczema doesn't just go away

- Children with eczema will always have dry skin
- Dry skin is usually due to skin inflammation
- Skin inflammation makes the skin itchy
- Itchy skin leads to scratching which cause skin damage and more itching
- Constant scratching can result in skin damage, this can be prevented with correct eczema treatment
- Moisturisers should be used every day, even when skin is clear

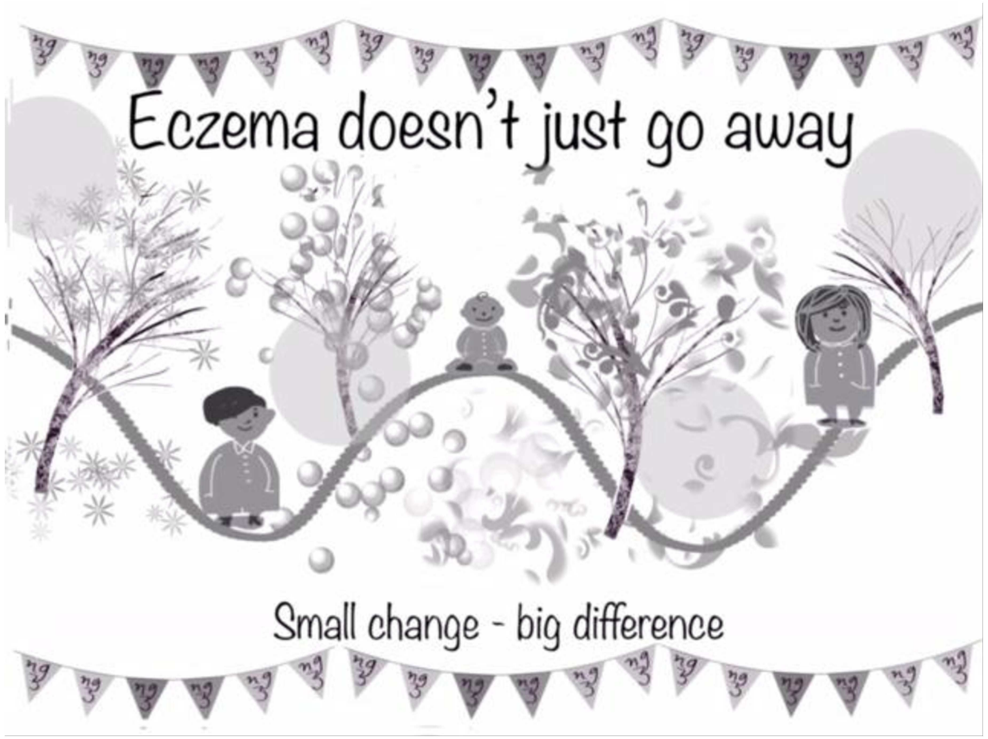

Moisturisers are for everyday

- Moisturisers (emollients) are the first line treatment for dry skin
- Emollients should be used every day, even when skin is clear
- Avoid soaps and bubble baths
- Best emollient is the one that suits your child
- You may need different emollients depending on skin dryness or time of year

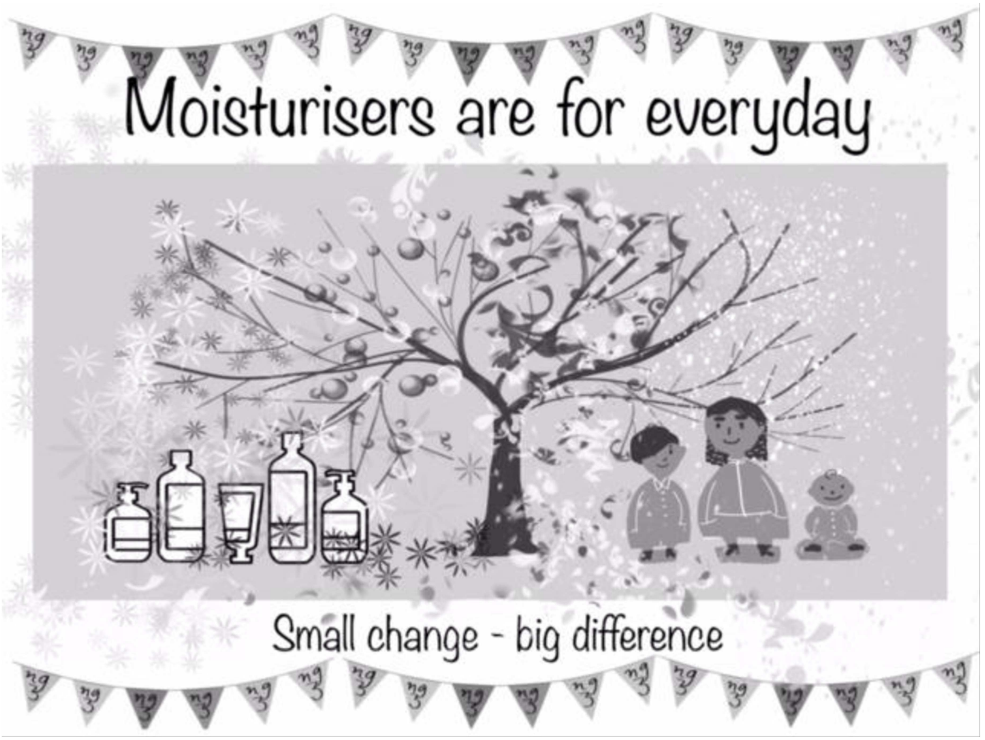

Steroid creams are okay when you need them

- Topical steroids are advised by the NHS as first-line treatments for eczema flares
- They are usually used for short treatment bursts
- It is important to use enough topical steroid, the finger tip unit can be a useful guide, which can be found in the leaflet in the steroid pack
- Topical steroids only need to be applied once a day.
- Topical steroids can have side effects but only if used for long periods and in large quantities in delicate areas such as the face. Your child is more likely to cause damage to their skin due to scratching

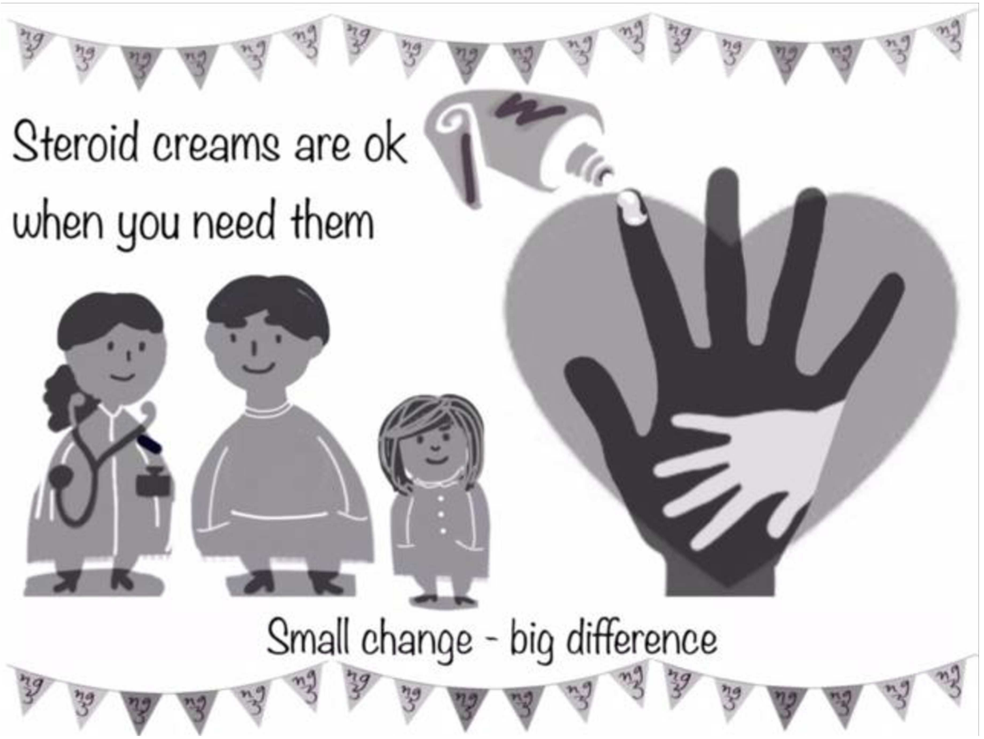

You know your child's eczema best

- Your pharmacist, health visitor, nurse or GP can help you to manage your child's eczema
- Book an appointment just to discuss your child's eczema
- Write down how you currently manage your child's eczema on a daily basis, use this to discuss how treatment can be improved
- Try to keep a record child's eczema and how it effects them
- If your child is prescribed topical steroids, ask how much, how often, how to apply and when to stop. Book a review appointment for 2 weeks

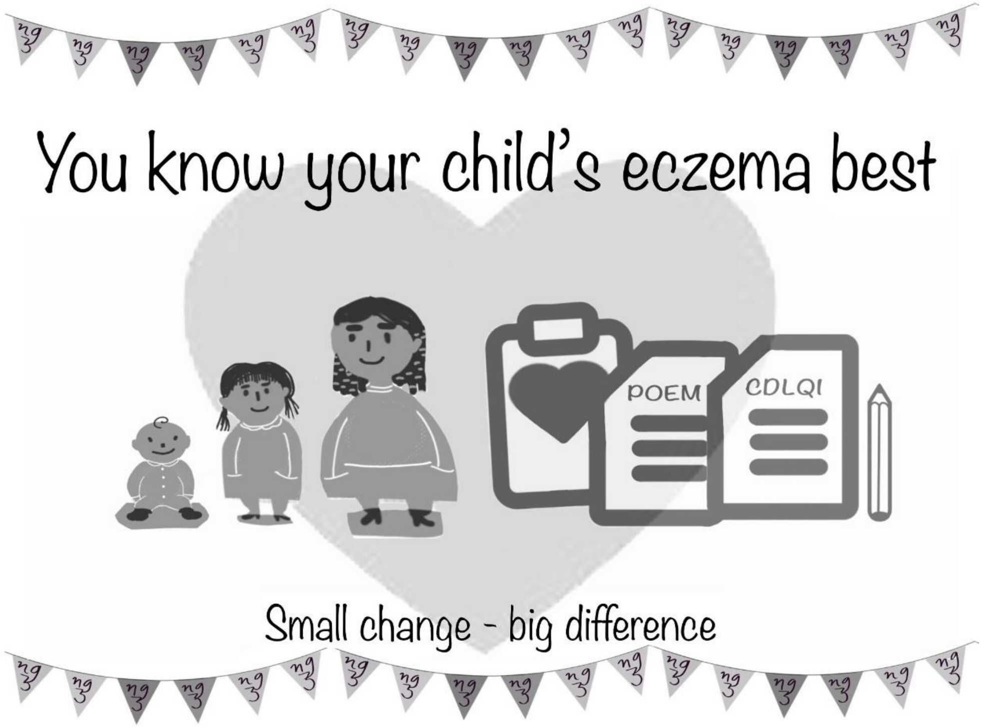

Supplement: Supplementary data [file bmjopen-2019-036520supp002.pdf]
