# Supplementary material for: Knowledge mobilisation: a UK co-creation study to devise strategies to amend lay and practitioner atopic eczema mindlines to improve consultation experiences and self-management practices in primary care
Source: BMJ Open. 2020 Sep 28;10(9):e036520. doi: 10.1136/bmjopen-2019-036520 (PMC7523205; doi:10.1136/bmjopen-2019-036520)
Supplement: Author's manuscript [file bmjopen-2019-036520.draft_revisions.pdf]

BMJ Open is committed to open peer review. As part of this commitment we make the peer review history of every article we publish publicly available.

When an article is published we post the peer reviewers' comments and the authors' responses online. We also post the versions of the paper that were used during peer review. These are the versions that the peer review comments apply to.

The versions of the paper that follow are the versions that were submitted during the peer review process. They are not the versions of record or the final published versions. They should not be cited or distributed as the published version of this manuscript.

BMJ Open is an open access journal and the full, final, typeset and author-corrected version of record of the manuscript is available on our site with no access controls, subscription charges or pay-per-view fees (http://bmjopen.bmj.com).

If you have any questions on BMJ Open's open peer review process please email info.bmjopen@bmj.com

BMJ Open

Knowledge mobilisation: a UK co-creation study to devise strategies to amend lay and practitioner atopic eczema mindlines to improve consultation experiences and self-management practices in primary care

Journal:	*BMJ Open*
Manuscript ID	bmjopen-2019-036520
Article Type:	Original research
Date Submitted by the Author:	18-Dec-2019
Complete List of Authors:	Cowdell, Fiona; Birmingham City University, Faculty of Health, Education and Life Sciences; University of Nottingham, Centre of Evidence Based Dermatology<br>Ahmed, Taheeya; University of Nottingham, Centre of Evidence Based Dermatology<br>Layfield , Carron; University of Nottingham, Centre of Evidence Based Dermatology
Keywords:	Eczema < DERMATOLOGY, Dermatology < INTERNAL MEDICINE, PRIMARY CARE, QUALITATIVE RESEARCH

SCHOLARONE™
Manuscripts

I, the Submitting Author has the right to grant and does grant on behalf of all authors of the Work (as defined in the below author licence), an exclusive licence and/or a non-exclusive licence for contributions from authors who are: i) UK Crown employees; ii) where BMJ has agreed a CC-BY licence shall apply, and/or iii) in accordance with the terms applicable for US Federal Government officers or employees acting as part of their official duties; on a worldwide, perpetual, irrevocable, royalty-free basis to BMJ Publishing Group Ltd ("BMJ") its licensees and where the relevant Journal is co-owned by BMJ to the co-owners of the Journal, to publish the Work in this journal and any other BMJ products and to exploit all rights, as set out in our licence.

The Submitting Author accepts and understands that any supply made under these terms is made by BMJ to the Submitting Author unless you are acting as an employee on behalf of your employer or a postgraduate student of an affiliated institution which is paying any applicable article publishing charge ("APC") for Open Access articles. Where the Submitting Author wishes to make the Work available on an Open Access basis (and intends to pay the relevant APC), the terms of reuse of such Open Access shall be governed by a Creative Commons licence – details of these licences and which Creative Commons licence will apply to this Work are set out in our licence referred to above.

Other than as permitted in any relevant BMJ Author's Self Archiving Policies, I confirm this Work has not been accepted for publication elsewhere, is not being considered for publication elsewhere and does not duplicate material already published. I confirm all authors consent to publication of this Work and authorise the granting of this licence.

For peer review only

BMJ Open Co-creation submitted Dec 2019

Knowledge mobilisation: a UK co-creation study to devise strategies to amend lay and practitioner atopic eczema mindlines to improve consultation experiences and self-management practices in primary care

Abstract

Objective: To devise strategies to amend lay and practitioner atopic eczema mindlines 'collectively reinforced, internalised tacit guidelines', to improve consultation experiences and self-management practices in primary care

Design: Co-creation workshops informed by the "Gold Standard" Coproduction Matrix

Setting: Conference centre in central England and via remote communication.

Participants: Lay people with, and parents of children with, atopic eczema, practitioners, a researcher and a facilitator (n=22).

Results: Eczema mindline amendment needs to address people and parents of children with the condition, practitioners and wider society in parallel. For lay people trust and "*realness*" of amendment activity was vital and practitioners wanted practical, locally relevant, hints and tips, tailored, 'no faff' approaches. To improve consultation experiences and self-management practices five key, consistent, evidence-based messages need to be instilled into eczema mindlines: i) eczema is more than just dry skin, ii) eczema doesn't just go away, iii) moisturisers are for every day, iv) steroid creams are okay when you need them and v) you know your child's eczema best.

Conclusion: This co-creation study provides original insights into *what* eczema knowledge should be mobilised, *who* needs to have this knowledge *how* this should be achieved to amend existing mindlines to improve consultation experiences and self-management practices in primary care. The remaining challenge is to refine, implement and evaluate the effectiveness of strategies developed to instil the five core messages and erase outdated or inaccurate information.

For peer review only

For peer review only - http://bmjopen.bmj.com/site/about/guidelines.xhtml

Strengths and limitations

- First co-creation study to examine strategies to amend lay-practitioner- social atopic eczema mindlines
- Diverse lay and practitioner co-creation group
- Only those with an existing interest in eczema joined the co-creation group

For peer review only

BMJ Open Co-creation submitted Dec 2019

Introduction

Atopic eczema (hereafter eczema) is a common burdensome long-term skin condition (1) with a high self-management demand (2). Evidence based treatment guidance is available, for example from the National Institute for Health and Clinical Excellence (3) and Clinical Knowledge Summaries (4). The mainstay of treatment is use of topical corticosteroids (TCS) at times of flare and regular, consistent application of emollients even when the skin appears healthy; in essence “getting control and keeping control” (5). Eczema management is 97% primary care based in the United Kingdom (6). Primary care consultations are often unsatisfactory for patients and practitioners alike (7, 8). Patients report practitioners with limited knowledge (9) and dismissive attitudes (10). Some practitioners describe uncertainly about optimum treatment (11); others regard eczema as simple to treat and perceive no need to change their current treatment “recipe” (12). A particular challenge is ensuring safe and appropriate use of topical corticosteroids. Steroid phobia is common (13) and reinforced at many levels (12, 14) potentially leading to under treatment, unwarranted suffering or treatment escalation (15) and wastage of prescribed medication (16). Eczema is a long-term condition, often with a high self-management demand. Long-term condition self-management is a policy imperative (17, 18, 19). Definitions of self-management vary (20) but involve ongoing efforts to maintain or improve health. Self-management interventions are designed to increase a person’s capacity, confidence and efficacy of to perform the necessary activities (21). Self-management of long-term skin conditions is notoriously challenging (22). Supportive interventions include bespoke programmes (23) and educational and psychological interventions (24, 25, 26). However, such offerings are not available to all, are costly and impact is inconsistent (23).

Knowledge mobilisation (KM) is essentially “moving knowledge to where it can be most useful” (27); it involves deliberative actions to create, disseminate and operationalise research and other forms of knowledge (28). KM is context specific (29), relational (30) and socially constructed (31). One approach to mobilising knowledge is through amending mindlines (12, 14, 32). Mindlines are

For peer review only - http://bmjopen.bmj.com/site/about/guidelines.xhtml

'collectively reinforced, internalised tacit guidelines' which underpin clinical decision making (33). They are built on a multifaceted combination of knowledge sources such as communication with colleagues and opinion leaders in the field and from personal tactic knowledge developed over time (33). Mindlines are founded in the work of Polyani (34) and Nonaka and Takeuchi (35); these authors recognise that knowledge is not necessarily conscious and explicit, but is in large part tacit and created from technical know-how and unconscious schemata. In practice this tacit knowledge is a far more influential than formal codified knowledge.

The seminal mindlines paper of Gabbay and Le May (36) has been cited 945 times to date. An extensive review a decade later (37) reveals little attention has been paid to condition specific mindlines or to a patient equivalent to mindlines, although Gabbay and le May (33) intimate their existence they do not develop this notion. Repeating the review search strategy reveals a continued absence of focus on patient mindlines with the exception of recent ethnographic work elucidating lay and practitioner eczema mindlines (12, 14, 32). These studies advocate improving eczema consultation experiences and self-management practices in primary care through a concerted effort amend eczema mindlines to increase shared knowledge, understanding and decision making. It is proposed that this may be achieved through parallel lay and practitioner mindline amendment.

Mindline amendment requires collaborative efforts from lay people, that is people with and parents of children with eczema, practitioners and researchers. Figure 1 illustrates the fundamentals of eczema mindlines i) lay people gain knowledge and beliefs from many sources, for example personal experience, family, friends and wider society, trial and error, online and from practitioners (14); ii) practitioner actions are underpinned by a belief that eczema is simple to treat "*the recipe doesn't change*" and that treatment options are limited by prescribing guidance, knowledge is accrued from early education, colleagues, practical experience and patients (12). Further work illuminates the inter-relationship between the two and therefore the need for efforts to amend

For peer review only - http://bmjopen.bmj.com/site/about/guidelines.xhtml

mindlines to be a shared venture which transcends lay-practitioner boundaries and instils shared and consistent understanding (32).

Insert Figure 1 here Lay (left side) and practitioner (right side) eczema mindlines and inter-relationship between the two.

Co-methodologies in health care are increasingly considered to be a "good thing". The language of "co" working remains contested (38, 39). There are a multitude of models but is it increasingly considered that co-working should involve lay people and professionals working as equals at every stage of the research process (39, 40). Essential questions when planning co-creation include *"who is participating, in what and for whose benefit"* (41).

In light of the prevalence of eczema, the high self-management demand, the challenges of primary care consultations and recent developments in understandings of lay and practitioners eczema mindlines (12, 14, 32), it seems prudent to investigate the way in which co-creation may be used to devise novel approaches to influencing these mindlines. The intention is to mobilise relevant, accurate, up to date and contextually appropriate knowledge to support positive primary care consultations and enable people with eczema to self-manage as effectively as possible.

Methods

Aim

To devise strategies to amend lay and practitioner eczema mindlines to improve consultation experiences and self-management practices in primary care

Objectives

1. To agree what a good consultation and effective self-management looks like
2. To identify
    a. *What* knowledge needs to be mobilised

For peer review only - http://bmjopen.bmj.com/site/about/guidelines.xhtml

BMJ Open Co-creation submitted Dec 2019

b. *Who* needs this knowledge

c. *How* should this knowledge be shared

Design

We employed a co-creation approach informed by the "Gold Standard" Coproduction Matrix (40) which comprises eight principles as summarised in Table 1.

Insert Table 1 here

Setting, co-creators and process

The co-creation group were recruited via a central England Higher Education Institute website with mass sharing via word of mouth and media posts, professional networks and an existing dermatology patient panel. Maximum variation purposive sampling (42) was applied in selecting co-creators of different age, gender and ethnicity, with experience of giving or receiving eczema care, ensuring a balance of lay and practitioner participants (n = 22). Potential co-creators were sent an information sheet. Those who agreed to take part received a copy of the original mindline publication of Gabbay and LeMay (36) and a copy of the lay and practitioner eczema mindline illustrations together with a brief explanation of how these had been developed and confirmed. The group met for a series of three face-to-face workshops and iterative email exchanges between April 2018 and March 2019. Workshops took place outside usual working hours at an accessible conference centre. The researcher (FC) and facilitator (CL), both of whom are experienced in co-creation, attended all three workshops. Practitioners attended workshop one, lay people with eczema, or who had children with eczema, attended workshop two and the full group attended workshop three. Following each workshop data were summarised and emailed to group members for discussion and approval. After the third workshop final refinement of ideas and terminology for key messages was achieved via email.

For peer review only - http://bmjopen.bmj.com/site/about/guidelines.xhtml

Although co-creators were nominally either lay or professional there was a degree of overlap, for example two of the practitioners had children with eczema and one lay person was also a practitioner. Demographic details are summarised in Table 2.

Insert Table 2 here

Data collection

Data collected included: facilitator notes written throughout the session, summaries of individual and group presentation information recorded on flip chart, checked by group members at the time of writing and artefacts, for example group member notes and products from activities. Details of workshops are summarised in Table 3.

Insert Table 3 here

Data analysis

Data collection and analysis were iterative processes (43). The researcher (FC) transcribed data from workshops and reviewed this as whole prior to summarising. FC and CL analysed the data summaries identifying key points and potential areas for mindline amendment as generated by the group. We paid attention to areas of agreement and disagreement between individuals and groups and circulated data summaries to group members for comment, supplementation and modification.

Reflexivity

A reflexive stance was maintained for the duration of the study with particular consideration being given to subjectivity and positioning of the researcher as a nurse with a particular interest in skin health; pre-understandings were consciously set aside (44).

For peer review only - http://bmjopen.bmj.com/site/about/guidelines.xhtml

For peer review only

Patient and Public Involvement

Lay people were involved in the development of the research question, planning and delivering the study and disseminating results.

Results

The co-creation group addressed four question. Each is discussed below with explanatory examples from the data.

i) *What makes a good eczema consultation in primary care?*

Lay group members suggested that a good consultation for them required a practitioner who understood the impact of eczema on their lives and had time to discuss these issues*, "usually not enough time to talk about the condition, how it effects life, more talking and understanding would be a good consultation".* Honesty about their knowledge was valued even when limited, provided they "*point us to useful information, the right direction and resources*". In parallel was the need for practitioners to respect individual's expertise in their own condition, "*I have an understanding, long-term and understand when I need help and something different*". Group members wanted to see the same practitioner when possible to avoid feeling *"sick and tired of saying the same story over and over again"*. They wanted practitioners to take eczema seriously, think of it as a long-term condition, "*to help manage 'long-term' rather than short-terms solution for flares*". Group members accepted their role in self-management with even the youngest stating the need for "*taking responsibility and sharing responsibility for condition ……….. makes you grow up*". Whilst embracing their contribution to long-term eczema control group members wanted to work with practitioners and to *"come to an understanding, work together, look at self-management … but … be there for support if needed".* They desired a sense of "*we can manage this, don't want to feel alone".*

For peer review only - http://bmjopen.bmj.com/site/about/guidelines.xhtml

For practitioners a good eczema consultation was characterised by empathy, with two-way dialogue between "*equals*" to generate shared understanding of eczema and treatment history asking "*tactful questions*" so that both parties understand and *"play the same tune, be on the same page".* Consistency in message from practitioners reduced misunderstandings and enhanced patient's confidence. They spoke of the long-term nature of eczema and the need to be "*blunt (honest!)*" about the need to "*explain long-term use of emollients to avoid flares*". Agreeing goals to "*get control and keep control*" was pivotal, with some advocating the provision of written information including action plans. Strategies to "*work with patients and motivate them to use emollients*" and "*emphasising long-term gain*" were promoted. It was important to find the "*right*" emollient. Being flexible to patient preference and experience was vital, albeit difficult within the confines of local prescribing guidelines.

ii) What should lay people and practitioners start, stop and continue to improve eczema consultations and self-management?

Co-creators identified the aspects of consultations that should be started, stopped and continued from both perspectives. Common threads included the need for a long-term approach to care, being prepared for the consultation, prioritising eczema, shared decision making and consistent use of topical treatments (see supplementary information 1 for more detail). Of note was that the group agreed that GPs were not necessarily the right practitioner to manage eczema and that more attention needs to be given to the role of other professionals, specifically community pharmacists, pharmacy counter assistants, nurses and health visitors.

iii) What are the priority areas for lay and practitioner eczema mindline amendment?

Distillation of data collected from the first two workshops and associated email exchanges identified seven key areas for mindline amendment: i) prioritise eczema, ii) manage eczema as a long-term condition, iii) prepare for each consultation, iv) be consistent with treatment, v) work together, vi)

For peer review only - http://bmjopen.bmj.com/site/about/guidelines.xhtml

get the right emollient and vii) use steroids appropriately. The facets of each area for mindline amendment are summarised in Table 4. Each area was viewed from both lay and practitioner perspectives.

Insert Table 4 here

Following a consensus activity the group prioritised three areas for mindline amendment; i) manage eczema as a long-term condition, ii) work together and iii) use topical steroids appropriately.

iv) How may lay and practitioner eczema mindlines best be amended?

The group highlighted challenges in modifying mindlines. These included the belief that eczema is not a priority for practitioners, some beliefs are deeply entrenched, and some reluctance to engage in shared decision making. In essence amendment activities suggested were multi-faceted. As each idea was proposed pros and cons were identified and many discounted as not feasible for reasons such as lack of resources, for example time and funding.

All agreed that interventions to amend the mindlines of either group in isolation would not be effective as they are inextricably linked. Co-creators identified the influence of wider social influences on beliefs about eczema care, for example in promulgating powerful messages, such as children “grow out of eczema” and the myth that topical steroid preparations are necessarily “dangerous”. For this reason they suggested that any attempt to amend mindlines would have to address all key players in parallel. For lay people trust and “*realness*” of amendment activity was vital and practitioners wanted practical, locally relevant, hints and tips, tailored, ‘no faff’ approaches.

Whilst amendment of knowledge was deemed important the group were unanimous in agreeing that providing information alone is not sufficient. There is also a need to redress power imbalances between patient and practitioner and promote shared understanding and decision making. With

For peer review only - http://bmjopen.bmj.com/site/about/guidelines.xhtml

this in mind, email exchanges with co-creators resulted in the three priorities being translated into five key, consistent, evidence based messages; i) eczema is more than just dry skin, ii) eczema doesn't just go away, iii) moisturisers are for every day, iv) steroid creams are okay when you need them and v) you know your child's eczema best. The latter was added as it was considered that initial mindline amendment activity should be targeted and, given the prevalence of childhood eczema, this was a reasonable focus. It also acknowledge the importance of parents' knowledge and expertise. Each priority is underpinned by a set of simple, illustrated messages (supplementary information 2). Given the need for consistency, clarity and straightforwardness of messages, at this stage we revisited existing evidence based resources including NICE guidance (3), CKS (4) and also consulted with the National Eczema Society to ensure consistency and fit with existing evidence base.

Co-creators worked with a range of resources to develop potential methods of amending lay and practitioner eczema mindlines; initial ideas for further development are summarised in Table 5.

Insert Table 5 here

Discussion

The aim of this co-creation study was to devise strategies to amend lay and practitioner eczema mindlines to improve consultation experiences and self-management practices in primary care. Particular emphasis was placed on defining *what* knowledge needs to be mobilised, w*ho* needs this knowledge and *how* should this knowledge be shared. These questions were answered through a series of structured co-creation workshops and virtual communications.

What knowledge needs to be mobilised? Although most lay people and practitioners know the fundamental "ingredients" of eczema care are TCS and emollients, it is knowledge about the

For peer review only - http://bmjopen.bmj.com/site/about/guidelines.xhtml

nuanced "recipe" for most effective use that needs to be amended. Alongside this the long-term nature of eczema and the need for mutual understanding between lay person and practitioner must be understood. *Who* needs this knowledge? Mindline amendment activity needs to be comprehensive, engaging people and parents of children with the condition, the full gamut of practitioners and people in wider society who may influence eczema care. *How* should this knowledge be shared? Importantly, knowledge alone is not sufficient to improve consultation experiences and self-management practices. There is a parallel need to change the balance of power in patient-practitioner relationships. These results are reflected in the five key messages to underpin eczema mindline amendment (see above).

This original study is one of the first to investigate how condition specific mindlines may best be amended across lay-practitioner-social boundaries. A robust approach to co-creation is demonstrated thorough adherence to the Gold Standards for Co-creation (40). In particular the process was well resourced, the group was diverse in terms of age, gender, ethnicity and expertise and was able to agree mutual aims and objectives and consistently and equally work together to achieve these. The co-creative process was reciprocal and progressive, steadily building on previous work to develop a sustainable approach to eczema mindline amendment. Reporting is in accordance with the consolidated criteria for reporting qualitative research (45).

Limitations are twofold, firstly only those with an interest in eczema were likely to take part, thus neglecting the many people who do not prioritise eczema care. Secondly, face-to-face co-creative time was limited, but this was mitigated by skilled facilitation, careful planning to ensure best use was made of available time and by using follow-up emails and conversations for clarification and development.

For peer review only - http://bmjopen.bmj.com/site/about/guidelines.xhtml

The need to address knowledge deficits, embrace key influencers across lay-practitioner-social boundaries and power imbalances led to the development of five key messages together with potential modes of delivery intended to bring about widespread changes in eczema mindlines. These are discussed in relation to existing literature. Findings from this present study reflect existing research which reports poor consultation experiences, practitioners with limited knowledge and apparent apathy and lack of priority given to eczema management by both some lay people and some practitioners (9-11). This mirrors the state of "gloom à deux" in which patients and practitioners share a perception of hopelessness (46), which is inimical to effective care. As with Gabbay and Le May (33), the present study points to the inter-relationship of patient-practitioner mindlines. Our findings are in accordance with and extend the findings of Gabbay and le May (33) who suggest that in a consultation two sets of mindlines converge as a single instantiation which is co-constructed during in the encounter. Our study reveals the need to manage this influence directly if eczema care is to improve, a more definitive call than the hint at social influences on health care and actions that subsequently occur (33).

To date little has been written about mindline amendment strategies however, there are elements of our co-created approaches that are congruent with existing literature. The need to provide clear, simple, "real" messages from trusted sources to a lay audience beyond those directly affected by eczema, but who may nevertheless influence use of treatments, can be grounded in the literature of social marketing (SM) in healthcare. SM amalgamates concepts from commercial marketing and social sciences; it goes beyond simply imparting knowledge widely and is intended to directly influence health care actions (47). SM in health care has been used internationally since the mid-1970s (48) and is considered a potential strategy to improve public health, for example in reduction of smoking and obesity (49). It embraces health communication techniques based on mass media. Messages can be mediated through other sources, in our case potentially practitioners and lay influencers. Communication approaches are varied and may include targeted message placement,

For peer review only - http://bmjopen.bmj.com/site/about/guidelines.xhtml

promotion, dissemination, and community outreach (50). Whilst evidence of the effectiveness of SM in healthcare remains sparse (51), it is argued that some elements have the potential to support lay mindline amendment, given the high prevalence of childhood eczema, the aim of changing behaviour and the need to get simple, consistent messages to a diverse range of people who have potential to influence treatment use (for example grandparents, friends and informal contacts).

Approaches to practitioner mindline amendment need to offer “no faff”, locally relevant, tailored hints and tips. This need is driven both by the low priority given to eczema and the increasingly heavy workloads of primary care practitioners (52, 53, 54, 55). Previous studies identify what is important to practitioners if messages are to influence their practice. Utility of information is measured by some in terms of relevance x validity ÷ work required to obtain it (56). Evidence suggests that to influence practice, knowledge must i) relate to the day-to-day challenge of having to manage this patient, at this time and in this situation (57), ii) be accessible, understandable and enable practitioners to make a “good enough” treatment decision and iii) fit with the particularities of the context (29, 58). Knowledge that addresses these points allows practitioners to transform it through contextual adroitness into knowledge-in-practice-in-context which enables good clinical care (59). Strategies to amend practitioner mindlines that take into account the needs outlined above may include, for example, use of aphorisms, that is, succinct sayings that offer advice and convey concentrated wisdom (60) or in a similar vein to “actionable nuggets”, snippets of practical and memorable information that can be readily used in everyday practice (61).

Although knowledge is essential, changing this facet of mindlines alone will not change eczema care. Power differentials between lay person and practitioner also need to be minimised. In agreement with existing literature our study concludes that knowledge-is-power (62) does not hold true in primary care eczema consultations. Whilst practitioners often have access to more clinical information, patients are frequently experts in their own condition (63). In common with existing

For peer review only - http://bmjopen.bmj.com/site/about/guidelines.xhtml

literature our group members agreed that when patients are engaged in healthcare decision making, outcomes (64) and levels of satisfaction (65) improve. The core messages for all we designed offer common language (66) and are intended to reduce “informational inequality” (67) and thus improve shared understanding and agreed, realistic plans of care.

Efforts to influence the mindlines of all types of health care provider are intended to support consistency of information. This approach also addresses power imbalances by widening the range of practitioners with whom lay people may choose to consult. Power imbalances are most frequently problematic in consultations with doctors, which begs the question whether they are best placed to provide eczema care. Eczema consultations with expert dermatology nurses are known to be highly valued by patients (68, 69, 70, 71). However, there are few primary care dermatology nurses in post. There are suggestions that community pharmacists and pharmacy counter assistants, who are often the first point of contact and who can offer advice independent of the pharmacist (72) are not yet being fully utilised (73, 74, 75).

Conclusion

This study, as with previous research, emphasises the need to improve eczema primary care consultation experiences and self-management practices. As previously noted approaches to date have predominantly been provision of educational and psychological interventions or the use of written action plans. The present study offers an alternative approach in mindline amendment in which simple, consistent, evidence based knowledge is shared across patient-practitioner-social boundaries to promote shared understanding and which, importantly, acknowledges role of influential members of the wider community. The challenge now is to take the five key messages, underpinned by hints and tips and convert them into a range of formats that can be shared amongst relevant parties. The impact of this intervention is intended to be revision or modification of mindlines achieved by adding reliable and useful knowledge and erasing outdated or inaccurate

For peer review only - http://bmjopen.bmj.com/site/about/guidelines.xhtml

information. Further research is needed to assess what bearing it has on primary care eczema consultation experiences and self-management practices.

Contribution statement
The authors made equal contributions to preparing this manuscript

Funding and disclaimer
Fiona Cowdell is funded by a National Institute for Health Research Knowledge Mobilisation Research Fellowship (KMRF-2015-04-004).

This article presents independent research funded by the National Institute for Health Research (NIHR). The views expressed are those of the authors and not necessarily those of the NHS, the NIHR or the Department of Health and Social Care.

Ethical approval
The study was approved by a university Research Ethics Committee.

Competing interests
None to declare

Acknowledgements
Thanks to all co-creators for their contribution to the group and in reviewing drafts of this manuscript and to Jay Nolan-Latchford for the mindline illustrations.

Data sharing statement
The datasets generated and/or analysed during the current study are not publicly available as they are not designed to be re-analysed by others but are available from the corresponding author on reasonable request.

For peer review only

References

1. Drucker AM, Wang AR, Li WQ, et al. The burden of atopic dermatitis: summary of a report for the National Eczema Association. *J. Investig. Dermatol* 2017;137(1):26-30.
2. Klinnert MD, Booster G, Copeland M, et al. Role of behavioral health in management of pediatric atopic dermatitis. *Ann Allerg Asthma Immunol* 2018;120(1):42-8.
3. National Institute for Health and Care Excellence. Atopic eczema in under 12s: diagnosis and management Clinical guideline [CG57] 2007. https://www.nice.org.uk/guidance/CG57 Accessed 17.12.19
4. National Institute for Health and Care Excellence. Clinical Knowledge Summary Eczema – atopic. 2018. https://cks.nice.org.uk/eczema-atopic Accessed 17.12.19
5. Tang TS, Bieber T, Williams HC. Are the concepts of induction of remission and treatment of subclinical inflammation in atopic dermatitis clinically useful? *J. Allergy Clin. Immunol* 2014; 133(6):1615-25.
6. Schofield JG, Grindlay D, Williams H. Skin conditions in the UK: a health care needs assessment. Centre of Evidence Based Dermatology, University of Nottingham, 2009.
7. Ali SF, Muhammad MP. Patient preference regarding their role in clinical decision making process: a systematic review. *Int J Nur Edu* 2014;6:192–5.
8. Horrocks S, Coast J. Patient choice: An exploration of primary care dermatology patients' values and expectations of care. *Qual Prim Care* 2007;15:185–93.
9. Santer M, Muller I, Yardley L, *et al*. Parents' and carers' views about emollients for childhood eczema: qualitative interview study. *BMJ Open* 2016;6:e011887.
10. Magin PJ, Adams J, Heading GS, et al. Patients with skin disease and their relationships with their doctors: a qualitative study of patients with acne, psoriasis and eczema. *Med J Australia* 2009;190(2):62-4.
11. Le Roux E, Powell K, Banks JP, *et al*. GPs' experiences of diagnosing and managing childhood eczema: a qualitative study in primary care. *Br J Gen Pract* 2018:bjgp18X694529
12. Cowdell F. Knowledge mobilisation: an ethnographic study of the influence of practitioner mindlines on atopic eczema self-management in primary care in the UK. *BMJ open* 2019a 1;9(7):e025220.
13. Hon KL, Kam WY, Leung TF, et al. Steroid fears in children with eczema. *Acta Paediatrica* 2006;95(11):1451-5.
14. Cowdell F. Knowledge mobilisation: an ethnographic study of the influence of lay mindlines on eczema self-management in primary care in the UK. *BMJ open* 2018;8(8):e021498.
15. Thomas KS, Stuart B, O'Leary CJ, et al. Validation of treatment escalation as a definition of atopic eczema flares. *PloS one* 2015;10(4):e0124770.
16. Smith SD, Hong E, Fearns S, *et al*. Corticosteroid phobia and other confounders in the treatment of childhood atopic dermatitis explored using parent focus groups. *Australas J Dermatol* 2010;51:168–74.
17. Miller WR, Lasiter S, Bartlett Ellis R, *et al*. Chronic disease self-management: a hybrid concept analysis. *Nurs Outlook* 2015;63:154–61.
18. Taylor SJC, Pinnock H, Epiphaniou E, *et al*. A rapid synthesis of the evidence on interventions supporting self-management for people with long-term conditions: PRISMS – Practical systematic RevIew of Self-Management Support for long-term conditions. *Health Services and Delivery Research* 2014;2:1–580.

For peer review only - http://bmjopen.bmj.com/site/about/guidelines.xhtml

19. Department of Health. A Mandate from the Government to NHS England. 2014. https://www.gov.uk/government/publications/nhs-mandate-2014-to-2015 Accessed 17.12.19
20. Lawn S, Schoo A. Supporting self-management of chronic health conditions: common approaches. *Patient Educ Couns* 2010;80:205–11.
21. Kennedy A, Bower P, Reeves D, *et al*. Implementation of self-management support for long term conditions in routine primary care settings: cluster randomised controlled trial. *BMJ* 2013;346:f2882.
22. Ersser SJ, Cowdell FC, Latter SM, *et al*. Self-management experiences in adults with mild-moderate psoriasis: an exploratory study and implications for improved support. *Br J Dermatol* 2010;163:1044–9.
23. Ridd MJ, King AJL, Le Roux E, *et al*. Systematic review of self-management interventions for people with eczema. *Br J Dermatol* 2017;177:719–34.
24. Ersser SJ, Cowdell F, Latter S, *et al*. Psychological and educational interventions for atopic eczema in children. *Cochrane Database Syst Rev* 2014:CD004054.
25. Bass AM, Anderson KL, Feldman SR. Interventions to increase treatment adherence in pediatric atopic dermatitis: a systematic review. *J Clin Med* 2015;4:231–42.
26. Pickett K, Loveman E, Kalita N, *et al*. Educational interventions to improve quality of life in people with chronic inflammatory skin diseases: systematic reviews of clinical effectiveness and cost-effectiveness. *Health Technol Assess* 2015;19:1–176.
27. Ward V. Why, whose, what and how? A framework for knowledge mobilisers. *Evid Policy* 2017;13:477–97.
28. Powell A, Davies HT, Nutley SM. Facing the challenges of research informed knowledge mobilization: 'Practising what we preach'? *Public Adm* 2017:1–17.
29. Jackson CL, Greenhalgh T. Co-creation: a new approach to optimising research impact? *Med J Aust* 2015;203:283–4.
30. Ferlie E, Crilly T, Jashapara A, *et al*. Knowledge mobilisation in healthcare: a critical review of health sector and generic management literature. *Soc Sci Med* 2012;74:1297–304.
31. Marshall M, Pagel C, French C, *et al*. Moving improvement research closer to practice: the researcher-in-Residence model. *BMJ Qual Saf* 2014;23:801–5.
32. Cowdell F. Knowledge mobilisation: an exploratory qualitative interview study to confirm and envision modification of lay and practitioner eczema mindlines to improve consultation experiences and self-management in primary care in the UK. *BMJ open.* 2019b;9(6):e028225.
33. Gabbay J, le May A. Practice-based evidence for healthcare: clinical mindlines. London: Routledge, 2011
34. Polyani M. The tacit dimension. Abingdon: Routledge & Kegan Paul, 1966.
35. Nonaka I, Takeuchi H. The knowledge creating company. New York: Oxford University Press, 1995.
36. Gabbay J, Le May A. Evidence based guidelines or collectively constructed "mindlines?" Ethnographic study of knowledge management in primary care. *Bmj* 2004;329(7473):1013.
37. Wieringa S, Greenhalgh T. 10 years of mindlines: a systematic review and commentary. *Implement Sci* 2015;10:45–55.
38. Flinders M, Wood M, Cunningham M. The politics of co-production: risks, limits and pollution. *Evidence & Policy: A Journal of Research, Debate and Practice* 2016;12(2):261-79.
39. INVOLVE NIHR. Co production. [Available from: http://www.invo.org.uk/current-work/co-production/ Accessed 17.12.19
40. Co:Create. The Coproduction matrix [Available from: https://www.wearecocreate.com/matrix/?rq=gold%20standard Accessed 17.12.19
41. Cornwall A. Unpacking 'Participation': models, meanings and practices. *Community Dev J* 2008;43(3):269-83.

For peer review only - http://bmjopen.bmj.com/site/about/guidelines.xhtml

42. Palinkas LA, Horwitz SM, Green CA, et al. Purposeful sampling for qualitative data collection and analysis in mixed method implementation research. *Admin Pol Ment Health* 2015;42(5):533-44.
43. Tracy SJ. Qualitative research methods: Collecting evidence, crafting analysis, communicating impact. John Wiley & Sons; 2019.
44. Berger R. Now I see it, now I don't: Researcher's position and reflexivity in qualitative research. *QRJ* 2015;15(2):219-34.
45. Tong A, Sainsbury P, Craig J. Consolidated criteria for reporting qualitative research (COREQ): a 32-item checklist for interviews and focus groups. *Int J Qual Health Care* 2007;19(6):349-357.
46. Dodd M. Motivational Interviewing Ideas for Peer Mentors. https://niatx.net/toolkits/system/IA_MIforPeerMentors.pdf Accessed 17.12.19
47. National Social Marketing Centre What is Social Marketing? https://www.thensmc.com/content/what-social-marketing-1 Accessed 17.12.19
48. Walsh DC, Rudd RE, Moeykens BA, Moloney TW. Social marketing for public health. *Health Aff* 1993;12(2):104-19.
49. Cheng H, Kotler P, Lee N, editors. Social marketing for public health: global trends and success stories. Jones & Bartlett Learning; 2011.
50. Evans WD. How social marketing works in health care. *BMJ* 2006;332(7551):1207-10.
51. Luca NR, Suggs LS. Theory and model use in social marketing health interventions. *J. Health Commun* 2013 Jan;18(1):20-40.
52. Doran N, Fox F, Rodham K, et al. Lost to the NHS: a mixed methods study of why GPs leave practice early in England. *Br J Gen Pract*. 2016;66(643):e128-35.
53. Ball J, Maben J, Griffiths P. Practice nursing: what do we know? British Journal of General Practice . https://bjgp.org/content/bjgp/65/630/10.full.pdf Accessed 17.12.19
54. Gregório J, Cavaco AM, Lapão LV. How to best manage time interaction with patients? Community pharmacist workload and service provision analysis. *RSAP* 2017;13(1):133-47.
55. Institute of Health Visiting 2019 Caseload https://ihv.org.uk/news_tag/caseload/ Accessed 17.12.19
56. Marie Cunningham A, Shirley A. Mindlines in a digital age. *Educ Prim Care* 2015;26(5):293-6.
57. Launer J. Guidelines and mindlines. *Postgrad Med J* 2015;91(1081):663-4.
58. Lau R, Stevenson F, Ong BN, et al. Achieving change in primary care—causes of the evidence to practice gap: systematic reviews of reviews. *Implement Sci* 2015;11(1):40.
59. Gabbay J, le May A. Mindlines: making sense of evidence in practice. BJGP https://bjgp.org/content/66/649/402.short Accessed 17.12.19
60. Levine D, Bleakley A. Maximising medicine through aphorisms. *Med Educ* 2012;46(2):153-62.
61. McColl MA, Aiken A, Smith K, et al. Actionable nuggets: knowledge translation tool for the needs of patients with spinal cord injury. *CFP* 2015;61(5):e240-8.
62. Snider A. Francis Bacon and the authority of aphorism. *Prose Studies* 1988;11(2):60-71.
63. O'Neill N, Mitchell G, Twycross A. The expert patient. Evidence-Based Nursing. 2016 Feb 16:ebnurs-2016. http://dx.doi.org/10.1136/eb-2016-102325 Accessed 17.12.19
64. Shortell SM, Poon BY, Ramsay PP, et al. A multilevel analysis of patient engagement and patient-reported outcomes in primary care practices of accountable care organizations. *J. G. Intern. Med* 2017;32(6):640-7.
65. Rieckmann P, Centonze D, Elovaara I, et al. Unmet needs, burden of treatment, and patient engagement in multiple sclerosis: A combined perspective from the MS in the 21st Century Steering Group. *Mult Scler Relat Dis* 2018;19:153-60.
66. Killian L, Coletti M. The role of Universal Health Literacy Precautions in minimizing "medspeak" and promoting shared decision making. *AMA J. Ethics* 2017;19(3):296-303.

For peer review only - http://bmjopen.bmj.com/site/about/guidelines.xhtml

67. Kashaf M.S. & McGill E. Does Shared Decision Making in Cancer Treatment Improve Quality of Life? A Systematic Literature Review. *Med Decis Making* 2015;35(8):1037-1048.
68. Schuttelaar ML, Vermeulen KM, Drukker N, et al. A randomized controlled trial in children with eczema: nurse practitioner vs. dermatologist. *Br J Dermatol* 2010;162(1):162-70.
69. Cork MJ, Britton J, Butler L, et al. Comparison of parent knowledge, therapy utilization and severity of atopic eczema before and after explanation and demonstration of topical therapies by a specialist dermatology nurse. *Br J Dermatol* 2003;149(3):582-9.
70. Chinn DJ, Poyner T, Sibley G. Randomized controlled trial of a single dermatology nurse consultation in primary care on the quality of life of children with atopic eczema. *Br J Dermatol* 2002;146(3):432-9.
71. Moore E, Williams A, Manias E, Varigos G. Nurse-led clinics reduce severity of childhood atopic eczema: a review of the literature. *Br J Dermatol* 2006;155 (6):1242-8.
72. Tucker R, Stewart D. An exploratory study of the views of community pharmacy staff on the management of patients with undiagnosed skin problems. *Int J Pharm Pract* 2015;23(6):390-8.
73. Carr A, Patel R, Jones M, Suleman A. A pilot study of a community pharmacist intervention to promote the effective use of emollients in childhood eczema. *Pharm J* 2007;278:17.
74. Ward PR, Bissell P, Noyce PR. Medicines counter assistants: roles and responsibilities in the sale of deregulated medicines. *Int J Pharm Pract* 1998;6(4):207-15.
75. Rutter P. Role of community pharmacists in patients' self-care and self-medication. *Integr Pharm Res Prac* 2015;4:57.

For peer review only

- Holistic: Coproduction should happen at every stage
- Resourced: Meaningful and effective coproduction deserves and requires sufficient resource
- Transparent: Coproduction should have a clear and transparent remit i.e.: overall aims, limitations, expectations and commitment
- Inclusive: Coproduction should involve a wide range of people (e.g.: practitioners, customers, future users, the wider community), capturing individual and differing views
- Iterative: Coproduction should be reciprocal, repeated, and progressive, always adapting and building upon what came before
- Positive: Coproduction should be mutually beneficial and an overall positive experience
- Equal: Each participant and their contribution should be valued equally
- Sustainable: Meaningful coproduction should have a genuine sustainable impact on the project

Table 1: Eight principles of the "Gold Standard" Coproduction Matrix

For peer review only

For peer review only - http://bmjopen.bmj.com/site/about/guidelines.xhtml

BMJ Open Co-creation submitted Dec 2019

Role	Gender	Age range
Lay person	F	35-44
Lay person	M	Under 16
Lay person	F	Under 16
Lay person	M	Over 55
Lay person	F	Over 55
Lay person	F	25-34
Lay person	F	45-54
Lay person	F	25-34
Lay person	F	45-54
Lay person	F	35-44
Dermatology specialist nurse	F	45-54
General practitioner	F	25-34
General practitioner trainee	M	25-34
Practice nurse	F	45-54
Medical student	F	17-24
Pharmacist	M	25-34
General practitioner trainee	F	25-34
Primary care nurse	F	45-54
Pharmacist	M	45-54
Pharmacy counter assistant	F	35-44
Facilitator	F	45-54
Researcher, facilitator and nurse	F	45-54

Table 2: Demographic details of the co-creation group

For peer review only

For peer review only - http://bmjopen.bmj.com/site/about/guidelines.xhtml

BMJ Open Co-creation submitted Dec 2019

Workshop	Attendees	Time	Activity
1	Practitioners, researcher and facilitator	2.5 hours	• Introductions and clarification of purpose of workshop.<br>• Discussion and questions about the circulated article and eczema mindlines illustrations.<br>• Selection from a choice of ordinary postcards that co-creators thought represented a "good" eczema consultation; each person briefly presented their thoughts.<br>• Small group exercise to identify behaviours that patients and practitioners should start, stop and continue to bring about an improvement in eczema consultations and self-management.<br>• Identification of initial priorities for mindline amendment.
2	Lay people, researcher and facilitator	3 hours	• As above
3	Lay people, practitioners, researcher and facilitator	One day	• Introductions and clarification of purpose of workshop<br>• Recap and discussion about the process and outcomes of workshops one and two.<br>• Co-creators individually reviewed priorities for mindline amendment derived from sessions 1 and 2 (Table 4) and ranked these in order of which they considered most likely to lead to improvement in consultation experience and self-management.<br>• Individuals worked in three mixed subgroups to identify their top three priorities.<br>• Whole group reconvened and each subgroup presented their rationale and choice for their top three after which, through consensus activity, a final three priorities for action were agreed.<br>• Subgroups worked with a range of creative resources to contemplate how lay and practitioner eczema mindlines may best be amended.

Table 3: Details of co-creation workshops

For peer review only - http://bmjopen.bmj.com/site/about/guidelines.xhtml

BMJ Open Co-creation submitted Dec 2019

**Priorities for mindline amendment**	**Facets**
Prioritise eczema	Be attentive, make eczema the primary reason for consultation, offer or go for follow-up
Manage eczema as a long-term condition	Educate, explain, avoid quick fixes, don't expect a cure, "get control, keep control"
Prepare for each consultation	Be aware of patient history, offer facts and good explanation, plan what to say, know what you want to achieve
Be consistent with treatment	Be concordant, know products and how to use them, agree realistic regimens, be truthful, don't waste, try over the counter products, understand local formulary
Work together	Listen and question, acknowledge expertise, understand burden of treatment, plan, get control, keep control
Get the right emollient	Be familiar with products, offer choice, agree feasible regimen
Use topical steroids appropriately	Understand risk and benefits, use for flares, use the best product for the optimum time

Table 4: Priorities for and facets of eczema mindline amendment

For peer review only

- Holding ‘pop ups’ of eczema information in places not currently targeted
- Having simple information available in skin care sections of supermarkets
- Targeting information at ‘Health Living Pharmacies’
- Using “Steroid Sam the emoji” in information to illustrate topical steroids in a more positive light
- Making more use of the POEM (Patient Oriented Eczema Measure) / Children’s Dermatology Life Quality Index to so parents can be more confident in “proving” impact of eczema during consultations
- Offering a “recipe” for better skin
- Build on existing resources for example Dr Ranj
  https://www.youtube.com/watch?v=lkIekfkoSbI
  https://www.youtube.com/watch?v=xM6XaL0gaho

Table 5: Initial ideas for mindline amendment strategies

For peer review only

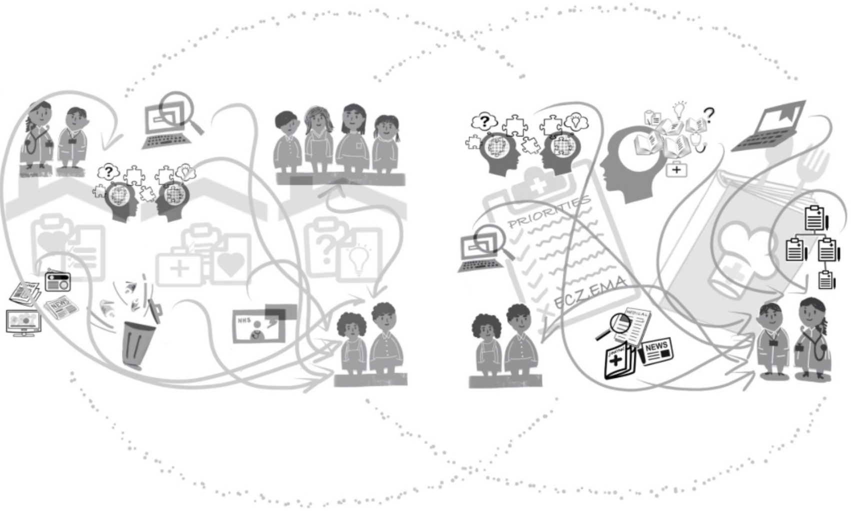

Lay (left side) and practitioner (right side) eczema mindlines and inter-relationship between the two.

394x295mm (132 x 132 DPI)

**Practitioners suggested that lay people should ………….**		
**Start**	**Continue**	**Stop**
Adhering to regular emollient use	Asking questions and challenging the practitioner	Having eczema as a secondary reason for consultation
Be truthful about their concerns	Going to see their practitioner	Stopping regular emollients
Being forceful if not being listened to, asking questions	Asking for advice	Agreeing to care plans they could or would not follow
Committing to the agreed plan of care	Being invested in their skin, wanting to look after it	Waiting for flares before coming for review
Accessing the evidence not the gossip	Be honest with self and practitioner	Not persevering with advice
Realising it's a chronic condition	Listening, writing it down	Using known "triggers"
Finding the right emollient for them	Owning their bodies	Listening to the ill informed
**Practitioners suggested that practitioners should ………….**		
**Start**	**Continue**	**Stop**
Recognising their limitations, accessing education and referring for help	Educating, explaining, supporting and reinforcing messages	Dismissing it as "only" eczema
Explain *how* and *when* to use treatments, monitor side effects and efficacy and give practical tips on useage	Validating concerns	Prescribing products that are impossible to use
Advising about over-the-counter products	Prescribing sufficient emollient prescribing	Viewing all patients as the same
Listening and empathising	Reiterating it's a long-term condition	Thinking eczema is one issue, it effects more than just skin
Signpost or refer for further information or treatment	Reassuring, there will be flares and good times	Under-prioritising how eczema affects patient's lives
Explaining chronicity, encourage regular review	Provide treatment options	Not looking at patient history relevant to consultations
Be familiar with products and provide patients with a selection of emollients so they find the best for them	Myth busting	Presenting cheapest option that doesn't fulfil patient need
Lobbying for products people can use	Recognising impact of eczema on quality of life	Underusing steroids
**Lay people suggested that lay people should ………….**		
**Start**	**Continue**	**Stop**
Preparing for the consultation, what do I want to achieve?	Providing updates	Assuming they know all the answers
Talking to them about long-term care	Choosing the right practitioner for the problem	Expecting a cure
Asking confidently, robustly for what you need and expecting questions to be answered	Trying what you have been asked to try irrespective of first impressions	Holding back, feel confident to say what needs to be said
**Lay people suggested that practitioners should ………….**		
**Start**	**Continue**	**Stop**

For peer review only - http://bmjopen.bmj.com/site/about/guidelines.xhtml

Being more approachable to patients knowledge, requests and understanding	Prescribing what works for the patient	Fobbing us off
Explain *how* to apply emollients and corticosteroids, explain the options	Prescribing as the patient wants	Trying to manage it in primary care if you have reached your limit
Being more realistic about the burden of treatment	Educating themselves clinically	Trivialising and dismissing the condition
Referring patients when prescribed treatments are not sufficient	Being open minded, each patient is unique	Telling us not to scratch
Having a better understanding of medication that's available	Educate themselves about the impact on lives	Treating just our skin, treat the whole person
Knowing the person's eczema history	Continue to educate themselves clinically	Thinking about cost so much
Be equipped with patient history	Value the patient's experience	Making false promises

Supplementary information 1: Practices that lay people and practitioners should start, stop and continue to improve consultation experiences.

For peer review only

Eczema is more than just dry skin

- 1 in 5 babies and children have atopic eczema
- A tendency to atopic eczema often runs in families
- There is no one cause
- Faulty skin barrier means the skin is dry, itchy and inflamed
- Eczema is made worse by ‘triggers’ which can be difficult to avoid

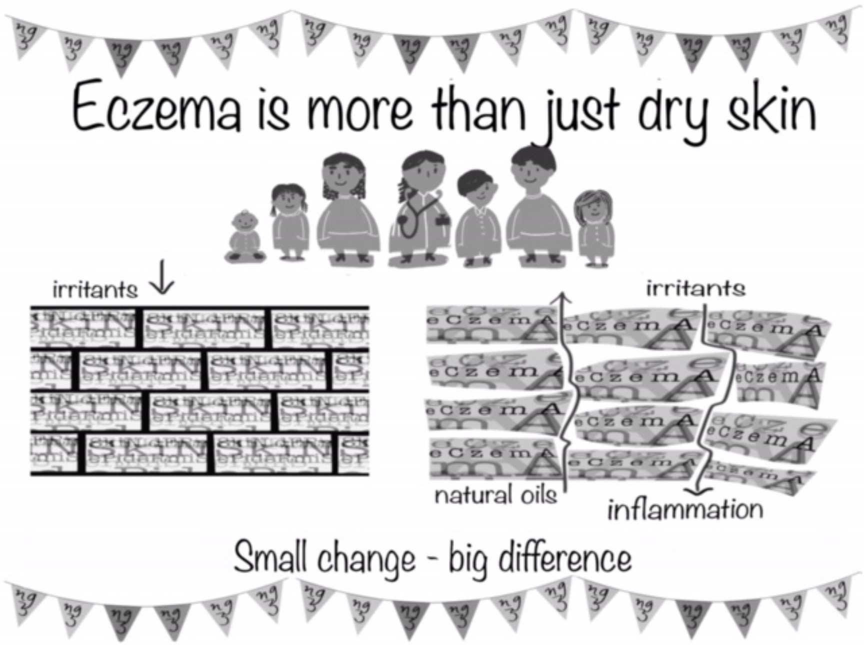

Eczema doesn't just go away

- Children with eczema will always have dry skin
- Dry skin is usually due to skin inflammation
- Skin inflammation makes the skin itchy
- Itchy skin leads to scratching which cause skin damage and more itching
- Constant scratching can result in skindamage, this can be prevented with correct eczema treatment
- Moisturisers should be used every day, even when skin is clear

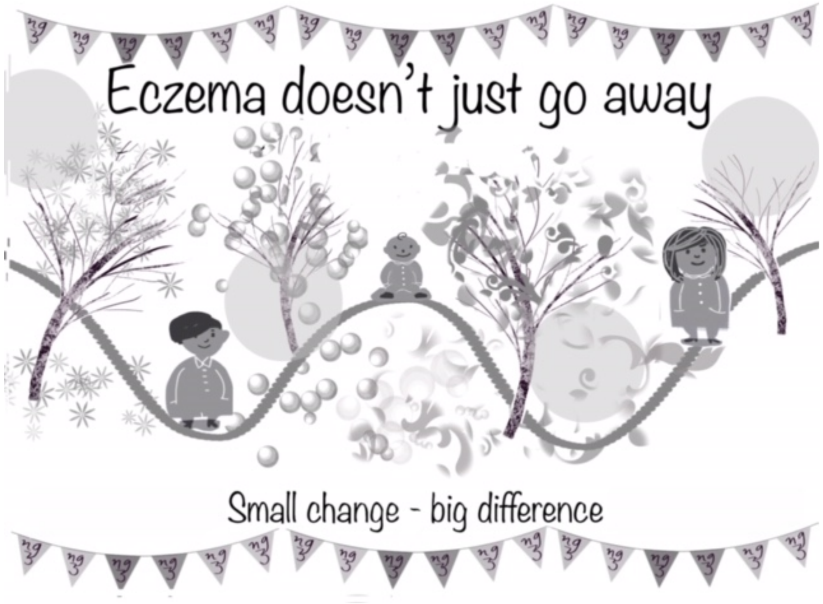

Moisturisers are for everyday

- Moisturisers (emollients) are the first line treatment for dry skin
- Emollients should be used every day, even when skin is clear
- Avoid soaps and bubble baths
- Best emollient is the one that suits your child
- You may need different emollients depending on skin dryness or time of year

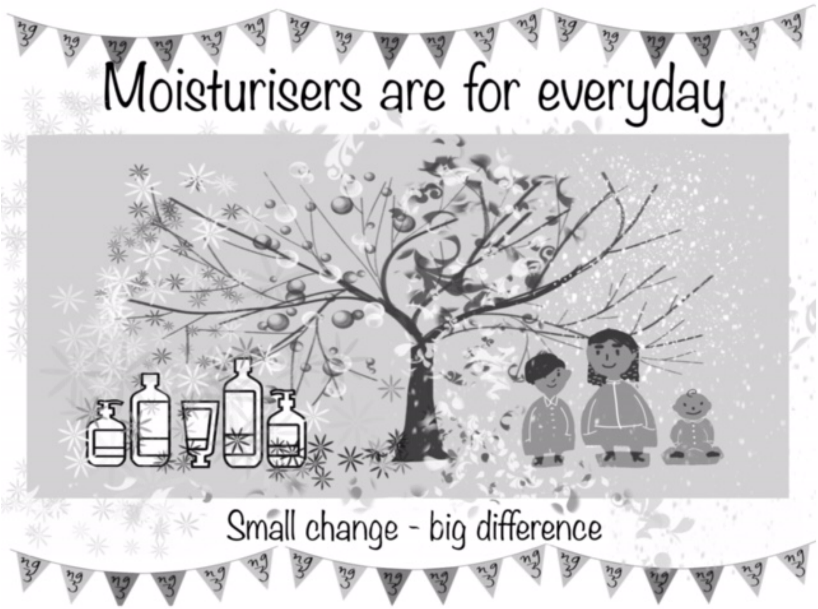

Steroid creams are okay when you need them

- Topical steroids are advised by the NHS as first-line treatments for eczema flares
- They are usually used for short treatment bursts
- It is important to use enough topical steroid, the finger tip unit can be a useful guide, which can be found in the leaflet in the steroid pack
- Topical steroids only need to be applied once a day
- Topical steroids can have side effects but only if used for long periods and in large quantities in delicate areas such as the face. Your child is more likely to cause damage to their skin due to scratching

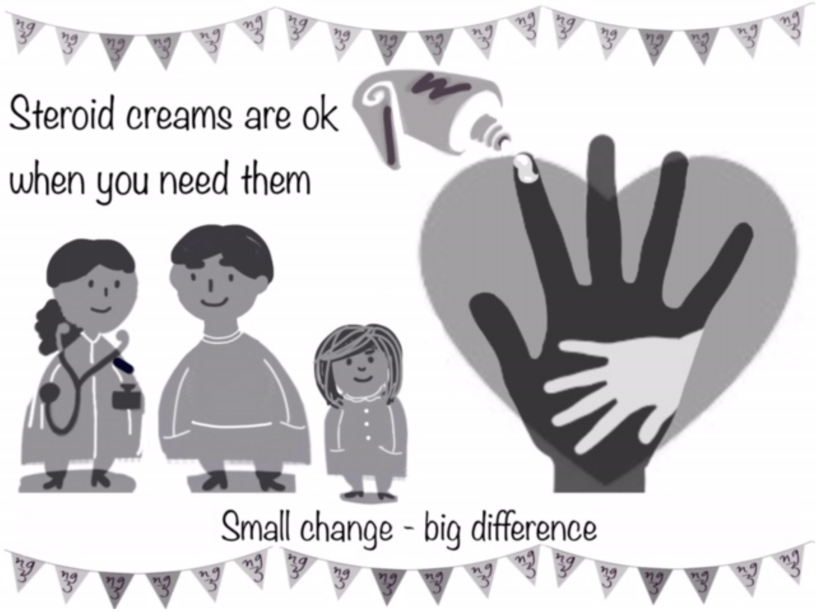

You know your child's eczema best

- Your pharmacist, health visitor, nurse or GP can help you to manage your child's eczema
- Book an appointment just to discuss your child's eczema
- Write down how you currently manage your child's eczema on a daily basis, use this to discuss how treatment can be improved
- Try to keep a record child's eczema and how it effects them
- If your child is prescribed topical steroids, ask how much, how often, how to apply and when to stop. Book a review appointment for 2 weeks

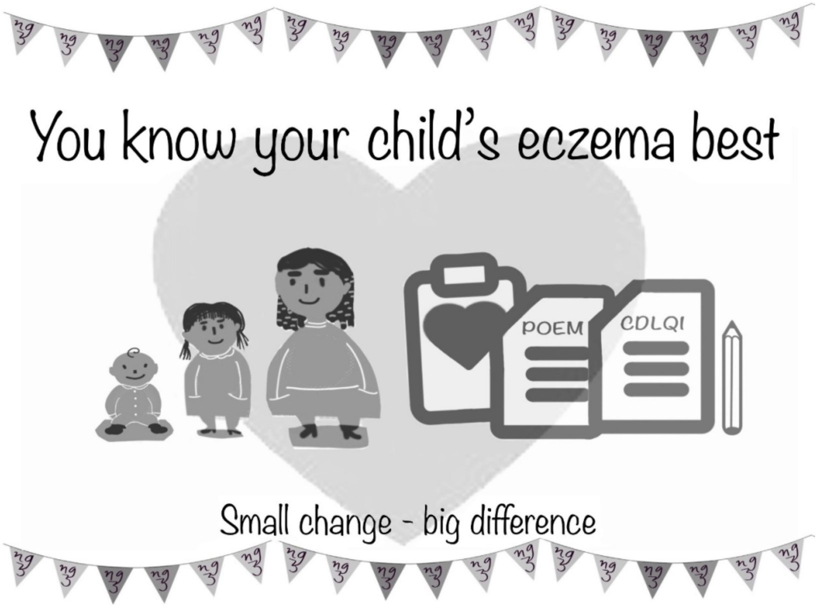

Title Page

Article title: Knowledge mobilisation: a UK co-creation study to devise strategies to amend lay and practitioner atopic eczema mindlines to improve consultation experiences and self-management practices in primary care

Affiliations

Corresponding author

Fiona Cowdell DProf, RN
Faculty of Health, Education and Life Sciences, Birmingham City University
Centre of Evidence Based Dermatology, University of Nottingham

223 Ravensbury House, Westbourne Road, Edgbaston, Birmingham, B15 3TN, UK
Telephone: +44 (0)121 300 4345
Email: Fiona.cowdell@bcu.ac.uk
orcid.org/0000-0002-9355-8059

Taheeya Ahmed
Centre of Evidence Based Dermatology, University of Nottingham

Carron Layfield
Centre of Evidence Based Dermatology, University of Nottingham

Word count: 3851

Number of figures and tables: one figure, five tables, two supplementary information

Key words: eczema, co-creation, knowledge mobilisation, mindlines, primary care, self-management

For peer review only

BMJ Open co-creation eczema mindlines COREQ 17.12.19

COREQ Statement

Knowledge mobilisation: An ethnographic study of the influence of practitioner mindlines on eczema self-management in primary care in the United Kingdom

Statement	Page no
**Domain 1: Research team and reflexivity**<br>*Personal Characteristics*<br>1. Interviewer/facilitator Which author/s conducted the interview or focus group?<br>Co-creation workshops were led by FC and CL	6
2. Credentials What were the researcher's credentials? E.g. PhD, MD<br>DProf, RN	Title page
3. Occupation What was their occupation at the time of the study?<br>Professor of Nursing and Health Research	Title page
4. Gender Was the researcher male or female?<br>Female	Title page
5. Experience and training What experience or training did the researcher have?<br>I have extensive experience in qualitative research.	Title page
Relationship with participants<br>6. Relationship established Was a relationship established prior to study commencement?<br>I established rapport with each co-creator through the recruitment process and at the beginning of each group	6 and Table 3
7. Participant knowledge of the interviewer<br>What did the participants know about the researcher? e.g. personal goals, reasons for doing the research<br>I provided an information sheet, explained about myself and the study and answered questions.	6
8. Interviewer characteristics What characteristics were reported about the interviewer/facilitator? e.g. Bias, assumptions, reasons and interests in the research topic<br>Participants were aware, and it is reported in the manuscript, that I am a Registered Nurse with an interest in how eczema knowledge is developed and shared between patients and practitioners in primary care and that this was a publically funded study.	6
**Domain 2: study design**<br>*Theoretical framework*<br>9. Methodological orientation and theory<br>What methodological orientation was stated to underpin the study? e.g. grounded theory, discourse analysis, ethnography, phenomenology, content analysis<br>In the methods section I explain that this is a co-creation study investigating mindline amendment	6
Participant selection<br>10. Sampling How were participants selected? e.g. purposive, convenience, consecutive, snowball<br>Purposive sampling to ensure a mix of co-creators	6
11. Method of approach How were participants approached? e.g. face-to-face, telephone, mail, email<br>HEI website with mass sharing via word of mouth and media posts, professional networks and an existing dermatology patient panel	6
12. Sample size How many participants were in the study?<br>n = 22	6
13. Non-participation How many people refused to participate or dropped out? Reasons?<br>.	NA
Setting<br>14. Setting of data collection Where was the data collected? e.g. home, clinic, workplace<br>Accessible conference centre and via email / telephone	6
15. Presence of non-participants Was anyone else present besides the participants and researchers?	NA
16. Description of sample What are the important characteristics of the sample? e.g. demographic data, date<br>Interview participants were sampled by profession, gender and years in practice.	Table 2
Data collection<br>17. Interview guide Were questions, prompts, guides provided by the authors? Was it pilot tested?	NA

For peer review only

BMJ Open co-creation eczema mindlines COREQ 17.12.19

18. Repeat interviews Were repeat interviews carried out? If yes, how many?	NA
19. Audio/visual recording Did the research use audio or visual recording to collect the data?	No
20. Field notes Were field notes made during and/or after the interview or focus group?<br>Notes and flip charts used	7
21. Duration What was the duration of the interviews or focus group?	½ - 1 day<br>Table 3
22. Data saturation Was data saturation discussed?	NA
23. Transcripts returned Were transcripts returned to participants for comment and/or correction?<br>Data summaries were circulated to group members for comment, supplementation and modification	7
**Domain 3: analysis and findings**<br>*Data analysis*<br>24. Number of data coders How many data coders coded the data?<br>Data analysis was completed by FC and CL with the co-creation group	7
25. Description of the coding tree Did authors provide a description of the coding tree?<br>No	NA
26. Derivation of themes Were themes identified in advance or derived from the data?<br>Findings were derived from the data	7
27. Software What software, if applicable, was used to manage the data?<br>No	NA
28. Participant checking Did participants provide feedback on the findings?<br>Yes	7
Reporting<br>29. Quotations presented Were participant quotations presented to illustrate the themes / findings? Was each quotation identified? e.g. participant number<br>Quotations are provided	8-15
30. Data and findings consistent Was there consistency between the data presented and the findings?<br>Yes.	8-15
31. Clarity of major themes Were major themes clearly presented in the findings?<br>Yes.	8-15
32. Clarity of minor themes Is there a description of diverse cases or discussion of minor themes?<br>Yes,	8-15

For peer review only

BMJ Open

Knowledge mobilisation: a UK co-creation study to devise strategies to amend lay and practitioner atopic eczema mindlines to improve consultation experiences and self-management practices in primary care

Journal:	*BMJ Open*
Manuscript ID	bmjopen-2019-036520.R1
Article Type:	Original research
Date Submitted by the Author:	17-Jun-2020
Complete List of Authors:	Cowdell, Fiona; Birmingham City University, Faculty of Health, Education and Life Sciences; University of Nottingham, Centre of Evidence Based Dermatology<br>Ahmed, Taheeya; University of Nottingham, Centre of Evidence Based Dermatology<br>Layfield , Carron; University of Nottingham, Centre of Evidence Based Dermatology
**Primary Subject Heading**:	Dermatology
Secondary Subject Heading:	General practice / Family practice
Keywords:	Eczema < DERMATOLOGY, Dermatology < INTERNAL MEDICINE, PRIMARY CARE, QUALITATIVE RESEARCH

SCHOLARONE™
Manuscripts

I, the Submitting Author has the right to grant and does grant on behalf of all authors of the Work (as defined in the below author licence), an exclusive licence and/or a non-exclusive licence for contributions from authors who are: i) UK Crown employees; ii) where BMJ has agreed a CC-BY licence shall apply, and/or iii) in accordance with the terms applicable for US Federal Government officers or employees acting as part of their official duties; on a worldwide, perpetual, irrevocable, royalty-free basis to BMJ Publishing Group Ltd ("BMJ") its licensees and where the relevant Journal is co-owned by BMJ to the co-owners of the Journal, to publish the Work in this journal and any other BMJ products and to exploit all rights, as set out in our licence.

The Submitting Author accepts and understands that any supply made under these terms is made by BMJ to the Submitting Author unless you are acting as an employee on behalf of your employer or a postgraduate student of an affiliated institution which is paying any applicable article publishing charge ("APC") for Open Access articles. Where the Submitting Author wishes to make the Work available on an Open Access basis (and intends to pay the relevant APC), the terms of reuse of such Open Access shall be governed by a Creative Commons licence – details of these licences and which Creative Commons licence will apply to this Work are set out in our licence referred to above.

Other than as permitted in any relevant BMJ Author's Self Archiving Policies, I confirm this Work has not been accepted for publication elsewhere, is not being considered for publication elsewhere and does not duplicate material already published. I confirm all authors consent to publication of this Work and authorise the granting of this licence.

For peer review only

BMJ Open Co-creation submitted Dec 2019 (R1 16.6.20 Clean copy)

Title Page

Article title: Knowledge mobilisation: a UK co-creation study to devise strategies to amend lay and practitioner atopic eczema mindlines to improve consultation experiences and self-management practices in primary care

Affiliations
Corresponding author

Fiona Cowdell DProf, RN
Faculty of Health, Education and Life Sciences, Birmingham City University
Centre of Evidence Based Dermatology, University of Nottingham

223 Ravensbury House, Westbourne Road, Edgbaston, Birmingham, B15 3TN, UK
Telephone: +44 (0)121 300 4345
Email: Fiona.cowdell@bcu.ac.uk
orcid.org/0000-0002-9355-8059

Taheeya Ahmed
Centre of Evidence Based Dermatology, University of Nottingham

Carron Layfield
Centre of Evidence Based Dermatology, University of Nottingham

Word count: 3910

Number of figures and tables: one figure, five tables, two supplementary files

Key words: eczema, co-creation, knowledge mobilisation, mindlines, primary care, self-management

For peer review only

Knowledge mobilisation: a UK co-creation study to devise strategies to amend lay and practitioner atopic eczema mindlines to improve consultation experiences and self-management practices in primary care

Abstract

Objective: To devise strategies to amend lay and practitioner atopic eczema mindlines 'collectively reinforced, internalised tacit guidelines', to improve consultation experiences and self-management practices in primary care

Design: Co-creation workshops informed by the Co:Create Coproduction Matrix

Setting: Conference centre in central England and via remote communication.

Participants: Lay people with, and parents of children with, atopic eczema, practitioners, a researcher and a facilitator (n=22).

Results: Eczema mindline amendment needs to address people and parents of children with the condition, practitioners and wider society in parallel. For lay people trust and "*realness*" of amendment activity was vital and practitioners wanted practical, locally relevant, hints and tips, tailored, 'no faff' approaches. To improve consultation experiences and self-management practices five key, consistent, evidence-based messages need to be instilled into eczema mindlines: i) eczema is more than just dry skin, ii) eczema doesn't just go away, iii) moisturisers are for every day, iv) steroid creams are okay when you need them and v) you know your child's eczema best.

Conclusion: This co-creation study provides original insights into *what* eczema knowledge should be mobilised, *who* needs to have this knowledge *how* this should be achieved to amend existing mindlines to improve consultation experiences and self-management practices in primary care. The remaining challenge is to refine, implement and evaluate the effectiveness of strategies developed to instil the five core messages and erase outdated or inaccurate information.

For peer review only

Strengths and limitations

- First co-creation study to examine strategies to amend lay-practitioner- social atopic eczema mindlines
- Diverse lay and practitioner co-creation group
- Only those with an existing interest in eczema joined the co-creation group

For peer review only - http://bmjopen.bmj.com/site/about/guidelines.xhtml

Introduction

Atopic eczema (hereafter eczema) is a common burdensome long-term skin condition (1) with a high self-management demand (2). Evidence based treatment guidance is available, for example from the National Institute for Health and Clinical Excellence (3) and Clinical Knowledge Summaries (4). The mainstay of treatment is use of topical corticosteroids (TCS) at times of flare and regular, consistent application of emollients even when the skin appears healthy; in essence "getting control and keeping control" (5). Eczema management is 97% primary care based in the United Kingdom (6). Primary care consultations are often unsatisfactory for patients and practitioners alike (7, 8). Patients report practitioners with limited knowledge (9) and dismissive attitudes (10). Some practitioners describe uncertainly about optimum treatment (11); others regard eczema as simple to treat and perceive no need to change their current treatment "recipe" (12). A particular challenge is ensuring safe and appropriate use of topical corticosteroids. Steroid phobia is common (13) and reinforced at many levels (12, 14) potentially leading to under treatment, unwarranted suffering or treatment escalation (15) and wastage of prescribed medication (16). Eczema is a long-term condition, often with a high self-management demand. Long-term condition self-management is a policy imperative (17, 18, 19). Definitions of self-management vary (20) but involve ongoing efforts to maintain or improve health. Self-management interventions are designed to increase a person's capacity, confidence and efficacy of to perform the necessary activities (21). Self-management of long-term skin conditions is notoriously challenging (22). Supportive interventions include bespoke programmes (23) and educational and psychological interventions (24, 25, 26). However, such offerings are not available to all, are costly and impact is inconsistent (23).

Knowledge mobilisation (KM) is essentially "moving knowledge to where it can be most useful" (27); it involves deliberative actions to create, disseminate and operationalise research and other forms of knowledge (28). KM is context specific (29), relational (30) and socially constructed (31). One approach to mobilising knowledge is through amending mindlines (12, 14, 32). Mindlines are

For peer review only - http://bmjopen.bmj.com/site/about/guidelines.xhtml

'collectively reinforced, internalised tacit guidelines' which underpin clinical decision making (33). They are built on a multifaceted combination of knowledge sources such as communication with colleagues and opinion leaders in the field and from personal tactic knowledge developed over time (33). Mindlines are founded in the work of Polyani (34) and Nonaka and Takeuchi (35); these authors recognise that knowledge is not necessarily conscious and explicit, but is in large part tacit and created from technical know-how and unconscious schemata. In practice this tacit knowledge is a far more influential than formal codified knowledge.

The seminal mindlines paper of Gabbay and Le May (36) has been cited 945 times to date. An extensive review a decade later (37) reveals little attention has been paid to condition specific mindlines or to a patient equivalent to mindlines, although Gabbay and le May (33) intimate their existence they do not develop this notion. Repeating the review search strategy reveals a continued absence of focus on patient mindlines with the exception of recent ethnographic work by one of the authors of this paper (FC), elucidating lay and practitioner eczema mindlines (12, 14, 32). These studies advocate improving eczema consultation experiences and self-management practices in primary care through a concerted effort amend eczema mindlines to increase shared knowledge, understanding and decision making. It is proposed that this may be achieved through parallel lay and practitioner mindline amendment.

Mindline amendment requires collaborative efforts from lay people, that is people with and parents of children with eczema, practitioners and researchers. Figure 1 illustrates the fundamentals of eczema mindlines and shows the complexity of how knowledge is gained, from which sources and the inter-relationship between lay-practitioner-wider society mindlines. In essence, i) lay people gain knowledge and beliefs from many sources, for example personal experience, family, friends and wider society, trial and error, online and from practitioners (14); ii) practitioner actions are underpinned by a belief that eczema is simple to treat "*the recipe doesn't change*" and that

For peer review only - http://bmjopen.bmj.com/site/about/guidelines.xhtml

treatment options are limited by prescribing guidance, knowledge is accrued from early education, colleagues, practical experience and patients (12). Further work illuminates the inter-relationship between the two and therefore the need for efforts to amend mindlines to be a shared venture which transcends lay-practitioner boundaries and instils shared and consistent understanding (32).

Insert Figure 1 here Lay (left side) and practitioner (right side) eczema mindlines and inter-relationship between the two.

Co-methodologies in health care are increasingly considered to be a “good thing”. The language of “co” working remains contested (38, 39). There are a multitude of models but is it increasingly considered that co-working should involve lay people and professionals working as equals at every stage of the research process (39, 40). Essential questions when planning co-creation include *“who is participating, in what and for whose benefit”* (41).

In light of the prevalence of eczema, the high self-management demand, the challenges of primary care consultations and recent developments in understandings of lay and practitioners eczema mindlines (12, 14, 32), it seems prudent to investigate the way in which co-creation may be used to devise novel approaches to influencing these mindlines. The intention is to mobilise relevant, accurate, up to date and contextually appropriate knowledge to support positive primary care consultations and enable people with eczema to self-manage as effectively as possible.

Methods

Aim

To devise strategies to amend lay and practitioner eczema mindlines to improve consultation experiences and self-management practices in primary care

Objectives

1. To agree what a good consultation and effective self-management looks like

For peer review only - http://bmjopen.bmj.com/site/about/guidelines.xhtml

2. To identify

    a. *What* knowledge needs to be mobilised

    b. *Who* needs this knowledge

    c. *How* should this knowledge be shared

Design

We employed a co-creation approach informed by the "Gold Standard" Coproduction Matrix (40) which comprises eight principles as summarised in Table 1.

Insert Table 1 here

Setting, co-creators and process

The co-creation group were recruited via a central England Higher Education Institute website with mass sharing via word of mouth and media posts, professional networks and an existing dermatology patient panel. Maximum variation purposive sampling (42) was applied in selecting co-creators of different age, gender and ethnicity, with experience of giving or receiving eczema care, ensuring a balance of lay and practitioner participants (n = 22). Potential co-creators were sent an information sheet. Those who agreed to take part received a copy of the original mindline publication of Gabbay and LeMay (36) and a copy of the lay and practitioner eczema mindline illustrations together with a brief explanation of how these had been developed and confirmed. The group met for a series of three face-to-face workshops and iterative email exchanges between April 2018 and March 2019. Workshops took place outside usual working hours at an accessible conference centre. The researcher (FC) and facilitator (CL), both of whom are experienced in co-creation, attended all three workshops. Practitioners attended workshop one, lay people with eczema, or who had children with eczema, attended workshop two and the full group attended workshop three. Following each workshop data were summarised and emailed to group members

For peer review only - http://bmjopen.bmj.com/site/about/guidelines.xhtml

for discussion and approval. After the third workshop final refinement of ideas and terminology for key messages was achieved via email.

Although co-creators were nominally either lay or professional there was a degree of overlap, for example two of the practitioners had children with eczema and one lay person was also a practitioner. Demographic details are summarised in Table 2.

Insert Table 2 here

Data collection

Data collected included: facilitator notes written throughout the session, summaries of individual and group presentation information recorded on flip chart, checked by group members at the time of writing and artefacts, for example group member notes and products from activities. Details of workshops are summarised in Table 3.

Insert Table 3 here

Data analysis

Data collection and analysis were iterative processes (43). The researcher (FC) transcribed data from workshops and reviewed this as whole prior to summarising. FC and CL analysed the data summaries identifying key points and potential areas for mindline amendment as generated by the group. We paid attention to areas of agreement and disagreement between individuals and groups and circulated data summaries to group members for comment, supplementation and modification.

For peer review only - http://bmjopen.bmj.com/site/about/guidelines.xhtml

Reflexivity

A reflexive stance was maintained for the duration of the study with particular consideration being given to subjectivity and positioning of the researcher as a nurse with a particular interest in skin health; pre-understandings were consciously set aside (44).

Patient and Public Involvement

Lay people were involved in the development of the research question, planning and delivering the study and disseminating results.

Results

The co-creation group addressed four question. Each is discussed below with explanatory examples from the data.

i) What makes a good eczema consultation in primary care?

Lay group members suggested that a good consultation for them required a practitioner who understood the impact of eczema on their lives and had time to discuss these issues*, "usually not enough time to talk about the condition, how it effects life, more talking and understanding would be a good consultation".* Honesty about their knowledge was valued even when limited, provided they "*point us to useful information, the right direction and resources*". In parallel was the need for practitioners to respect individual's expertise in their own condition, "*I have an understanding, long-term and understand when I need help and something different*". Group members wanted to see the same practitioner when possible to avoid feeling *"sick and tired of saying the same story over and over again"*. They wanted practitioners to take eczema seriously, think of it as a long-term condition, "*to help manage 'long-term' rather than short-terms solution for flares*". Group members accepted their role in self-management with even the youngest stating the need for "*taking responsibility and sharing responsibility for condition ……….. makes you grow up*". Whilst embracing their contribution

For peer review only - http://bmjopen.bmj.com/site/about/guidelines.xhtml

to long-term eczema control group members wanted to work with practitioners and to *"come to an understanding, work together, look at self-management … but … be there for support if needed".* They desired a sense of "*we can manage this, don't want to feel alone".*

For practitioners a good eczema consultation was characterised by empathy, with two-way dialogue between "*equals*" to generate shared understanding of eczema and treatment history asking "*tactful questions*" so that both parties understand and *"play the same tune, be on the same page".* Consistency in message from practitioners reduced misunderstandings and enhanced patient's confidence. They spoke of the long-term nature of eczema and the need to be "*blunt (honest!)*" about the need to "*explain long-term use of emollients to avoid flares*". Agreeing goals to "*get control and keep control*" was pivotal, with some advocating the provision of written information including action plans. Strategies to "*work with patients and motivate them to use emollients*" and "*emphasising long-term gain*" were promoted. It was important to find the "*right*" emollient. Being flexible to patient preference and experience was vital, albeit difficult within the confines of local prescribing guidelines.

ii) What should lay people and practitioners start, stop and continue to improve eczema consultations and self-management?

Co-creators identified the aspects of consultations that should be started, stopped and continued from both perspectives. Common threads included the need for a long-term approach to care, being prepared for the consultation, prioritising eczema, shared decision making and consistent use of topical treatments (see supplementary information 1 for more detail). Of note was that the group agreed that GPs were not necessarily the right practitioner to manage eczema and that more attention needs to be given to the role of other professionals, specifically community pharmacists, pharmacy counter assistants, nurses and health visitors.

For peer review only

iii) What are the priority areas for lay and practitioner eczema mindline amendment?

Distillation of data collected from the first two workshops and associated email exchanges identified seven key areas for mindline amendment: i) prioritise eczema, ii) manage eczema as a long-term condition, iii) prepare for each consultation, iv) be consistent with treatment, v) work together, vi) get the right emollient and vii) use steroids appropriately. The facets of each area for mindline amendment are summarised in Table 4. Each area was viewed from both lay and practitioner perspectives.

Insert Table 4 here

Following a consensus activity the group prioritised three areas for mindline amendment; i) manage eczema as a long-term condition, ii) work together and iii) use topical steroids appropriately.

iv) How may lay and practitioner eczema mindlines best be amended?

The group highlighted challenges in modifying mindlines. These included the belief that eczema is not a priority for practitioners, some beliefs are deeply entrenched, and some reluctance to engage in shared decision making. In essence amendment activities suggested were multi-faceted. As each idea was proposed pros and cons were identified and many discounted as not feasible for reasons such as lack of resources, for example time and funding.

All agreed that interventions to amend the mindlines of either group in isolation would not be effective as they are inextricably linked. Co-creators identified the influence of wider social influences on beliefs about eczema care, for example in promulgating powerful messages, such as children “grow out of eczema” and the myth that topical steroid preparations are necessarily “dangerous”. For this reason they suggested that any attempt to amend mindlines would have to address all key players in parallel. For lay people trust and “*realness*” of amendment activity was vital and practitioners wanted practical, locally relevant, hints and tips, tailored, ‘no faff’ approaches.

For peer review only

Whilst amendment of knowledge was deemed important the group were unanimous in agreeing that providing information alone is not sufficient. There is also a need to redress power imbalances between patient and practitioner and promote shared understanding and decision making. With this in mind, email exchanges with co-creators resulted in the three priorities being translated into five key, consistent, evidence based messages; i) eczema is more than just dry skin, ii) eczema doesn't just go away, iii) moisturisers are for every day, iv) steroid creams are okay when you need them and v) you know your child's eczema best. The latter was added as it was considered that initial mindline amendment activity should be targeted and, given the prevalence of childhood eczema, this was a reasonable focus. It also acknowledge the importance of parents' knowledge and expertise. Each priority is underpinned by a set of simple, illustrated messages (supplementary information 2). Given the need for consistency, clarity and straightforwardness of messages, at this stage we revisited existing evidence based resources including NICE guidance (3), CKS (4) and also consulted with the National Eczema Society to ensure consistency and fit with existing evidence base.

Co-creators worked with a range of resources to develop potential methods of amending lay and practitioner eczema mindlines; initial ideas for further development are summarised in Table 5.

Insert Table 5 here

Discussion

The aim of this co-creation study was to devise strategies to amend lay and practitioner eczema mindlines to improve consultation experiences and self-management practices in primary care. Amending mindlines offers an entirely new approach to changing eczema care which goes beyond existing education interventions for patients and parents (for example 23, 24, 25, 26). It also addresses the identified challenges of primary care practitioners not prioritising their own eczema

For peer review only

education (12). In co-creation particular emphasis was placed on defining *what* knowledge needs to be mobilised, w*ho* needs this knowledge and *how* should this knowledge be shared. These questions were answered through a series of structured co-creation workshops and virtual communications.

What knowledge needs to be mobilised? Although most lay people and practitioners know the fundamental "ingredients" of eczema care are TCS and emollients, it is knowledge about the nuanced "recipe" for most effective use that needs to be amended. Alongside this the long-term nature of eczema and the need for mutual understanding between lay person and practitioner must be understood. *Who* needs this knowledge? Mindline amendment activity needs to be comprehensive, engaging people and parents of children with the condition, the full gamut of practitioners and people in wider society who may influence eczema care. *How* should this knowledge be shared? Importantly, knowledge alone is not sufficient to improve consultation experiences and self-management practices. There is a parallel need to change the balance of power in patient-practitioner relationships. These results are reflected in the five key messages to underpin eczema mindline amendment (see above).

This original study is one of the first to investigate how condition specific mindlines may best be amended across lay-practitioner-social boundaries. A robust approach to co-creation is demonstrated thorough adherence to the Gold Standards for Co-creation (40). In particular the process was well resourced, the group was diverse in terms of age, gender, ethnicity and expertise and was able to agree mutual aims and objectives and consistently and equally work together to achieve these. The co-creative process was reciprocal and progressive, steadily building on previous work to develop a sustainable approach to eczema mindline amendment. Reporting is in accordance with the consolidated criteria for reporting qualitative research (45).

For peer review only - http://bmjopen.bmj.com/site/about/guidelines.xhtml

Limitations are twofold, firstly only those with an interest in eczema were likely to take part, thus neglecting the many people who do not prioritise eczema care. Secondly, face-to-face co-creative time was limited, but this was mitigated by skilled facilitation, careful planning to ensure best use was made of available time and by using follow-up emails and conversations for clarification and development.

The need to address knowledge deficits, embrace key influencers across lay-practitioner-social boundaries and power imbalances led to the development of five key messages together with potential modes of delivery intended to bring about widespread changes in eczema mindlines. These are discussed in relation to existing literature. Findings from this present study reflect existing research which reports poor consultation experiences, practitioners with limited knowledge and apparent apathy and lack of priority given to eczema management by both some lay people and some practitioners (9-11). This mirrors the state of “gloom à deux” in which patients and practitioners share a perception of hopelessness (46), which is inimical to effective care. As with Gabbay and Le May (33), the present study points to the inter-relationship of patient-practitioner mindlines. Our findings are in accordance with and extend the findings of Gabbay and le May (33) who suggest that in a consultation two sets of mindlines converge as a single instantiation which is co-constructed during in the encounter. Our study reveals the need to manage this influence directly if eczema care is to improve, a more definitive call than the hint at social influences on health care and actions that subsequently occur (33).

To date little has been written about mindline amendment strategies however, there are elements of our co-created approaches that are congruent with existing literature. The need to provide clear, simple, “real” messages from trusted sources to a lay audience beyond those directly affected by eczema, but who may nevertheless influence use of treatments, can be grounded in the literature of social marketing (SM) in healthcare. SM amalgamates concepts from commercial marketing and

For peer review only - http://bmjopen.bmj.com/site/about/guidelines.xhtml

social sciences; it goes beyond simply imparting knowledge widely and is intended to directly influence health care actions (47). SM in health care has been used internationally since the mid-1970s (48) and is considered a potential strategy to improve public health, for example in reduction of smoking and obesity (49). It embraces health communication techniques based on mass media. Messages can be mediated through other sources, in our case potentially practitioners and lay influencers. Communication approaches are varied and may include targeted message placement, promotion, dissemination, and community outreach (50). Whilst evidence of the effectiveness of SM in healthcare remains sparse (51), it is argued that some elements have the potential to support lay mindline amendment, given the high prevalence of childhood eczema, the aim of changing behaviour and the need to get simple, consistent messages to a diverse range of people who have potential to influence treatment use (for example grandparents, friends and informal contacts).

Approaches to practitioner mindline amendment need to offer "no faff", locally relevant, tailored hints and tips. This need is driven both by the low priority given to eczema and the increasingly heavy workloads of primary care practitioners (52, 53, 54, 55). Previous studies identify what is important to practitioners if messages are to influence their practice. Utility of information is measured by some in terms of relevance x validity ÷ work required to obtain it (56). Evidence suggests that to influence practice, knowledge must i) relate to the day-to-day challenge of having to manage this patient, at this time and in this situation (57), ii) be accessible, understandable and enable practitioners to make a "good enough" treatment decision and iii) fit with the particularities of the context (29, 58). Knowledge that addresses these points allows practitioners to transform it through contextual adroitness into knowledge-in-practice-in-context which enables good clinical care (59). Strategies to amend practitioner mindlines that take into account the needs outlined above may include, for example, use of aphorisms, that is, succinct sayings that offer advice and convey concentrated wisdom (60) or in a similar vein to "actionable nuggets", snippets of practical and memorable information that can be readily used in everyday practice (61).

For peer review only - http://bmjopen.bmj.com/site/about/guidelines.xhtml

Although knowledge is essential, changing this facet of mindlines alone will not change eczema care. Power differentials between lay person and practitioner also need to be minimised. In agreement with existing literature our study concludes that knowledge-is-power (62) does not hold true in primary care eczema consultations. Whilst practitioners often have access to more clinical information, patients are frequently experts in their own condition (63). In common with existing literature our group members agreed that when patients are engaged in healthcare decision making, outcomes (64) and levels of satisfaction (65) improve. The core messages for all we designed offer common language (66) and are intended to reduce "informational inequality" (67) and thus improve shared understanding and agreed, realistic plans of care.

Efforts to influence the mindlines of all types of health care provider are intended to support consistency of information. This approach also addresses power imbalances by widening the range of practitioners with whom lay people may choose to consult. Power imbalances are most frequently problematic in consultations with doctors, which begs the question whether they are best placed to provide eczema care. Eczema consultations with expert dermatology nurses are known to be highly valued by patients (68, 69, 70, 71). However, there are few primary care dermatology nurses in post. There are suggestions that community pharmacists and pharmacy counter assistants, who are often the first point of contact and who can offer advice independent of the pharmacist (72) are not yet being fully utilised (73, 74, 75).

Conclusion

This study, as with previous research, emphasises the need to improve eczema primary care consultation experiences and self-management practices. As previously noted approaches to date have predominantly been provision of educational and psychological interventions or the use of written action plans. The present study offers an alternative approach in mindline amendment in which simple, consistent, evidence based knowledge is shared across patient-practitioner-social

For peer review only - http://bmjopen.bmj.com/site/about/guidelines.xhtml

boundaries to promote shared understanding and which, importantly, acknowledges role of influential members of the wider community. The challenge now is to take the five key messages, underpinned by hints and tips and convert them into a range of formats that can be shared amongst relevant parties. The impact of this intervention is intended to be revision or modification of mindlines achieved by adding reliable and useful knowledge and erasing outdated or inaccurate information. Further research is needed to assess what bearing it has on primary care eczema consultation experiences and self-management practices.

For peer review only

Contribution statement

FC conceived and designed the study. FC, TA and CL made substantial contributions to the acquisition, analysis and interpretation of data.

FC wrote the draft manuscript. TA and CL revised the draft critically for important intellectual content.

FC, TA and CL gave final approval of the version published.

FC, TA and CL agreed to be accountable for all aspects of the work in ensuring that questions related to the accuracy or integrity of any part of the work are appropriately investigated and resolved.

Funding and disclaimer

Fiona Cowdell is funded by a National Institute for Health Research Knowledge Mobilisation Research Fellowship (KMRF-2015-04-004).

This article presents independent research funded by the National Institute for Health Research (NIHR). The views expressed are those of the authors and not necessarily those of the NHS, the NIHR or the Department of Health and Social Care.

Ethical approval

The study was approved by the Birmingham City University, Health, Education and Life Sciences Faculty Academic Ethics Committee

Competing interests

None to declare

Acknowledgements

Thanks to all co-creators for their contribution to the group and in reviewing drafts of this manuscript and to Jay Nolan-Latchford for the mindline illustrations.

Data sharing statement

The datasets generated and/or analysed during the current study are not publicly available as they are not designed to be re-analysed by others but are available from the corresponding author on reasonable request.

For peer review only

References

1. Drucker AM, Wang AR, Li WQ, et al. The burden of atopic dermatitis: summary of a report for the National Eczema Association. *J. Investig. Dermatol* 2017;137(1):26-30.
2. Klinnert MD, Booster G, Copeland M, et al. Role of behavioral health in management of pediatric atopic dermatitis. *Ann Allerg Asthma Immunol* 2018;120(1):42-8.
3. National Institute for Health and Care Excellence. Atopic eczema in under 12s: diagnosis and management Clinical guideline [CG57] 2007. https://www.nice.org.uk/guidance/CG57 Accessed 17.12.19
4. National Institute for Health and Care Excellence. Clinical Knowledge Summary Eczema – atopic. 2018. https://cks.nice.org.uk/eczema-atopic Accessed 17.12.19
5. Tang TS, Bieber T, Williams HC. Are the concepts of induction of remission and treatment of subclinical inflammation in atopic dermatitis clinically useful? *J. Allergy Clin. Immunol* 2014; 133(6):1615-25.
6. Schofield JG, Grindlay D, Williams H. Skin conditions in the UK: a health care needs assessment. Centre of Evidence Based Dermatology, University of Nottingham, 2009.
7. Ali SF, Muhammad MP. Patient preference regarding their role in clinical decision making process: a systematic review. *Int J Nur Edu* 2014;6:192–5.
8. Horrocks S, Coast J. Patient choice: An exploration of primary care dermatology patients' values and expectations of care. *Qual Prim Care* 2007;15:185–93.
9. Santer M, Muller I, Yardley L, *et al*. Parents' and carers' views about emollients for childhood eczema: qualitative interview study. *BMJ Open* 2016;6:e011887.
10. Magin PJ, Adams J, Heading GS, et al. Patients with skin disease and their relationships with their doctors: a qualitative study of patients with acne, psoriasis and eczema. *Med J Australia* 2009;190(2):62-4.
11. Le Roux E, Powell K, Banks JP, *et al*. GPs' experiences of diagnosing and managing childhood eczema: a qualitative study in primary care. *Br J Gen Pract* 2018:bjgp18X694529
12. Cowdell F. Knowledge mobilisation: an ethnographic study of the influence of practitioner mindlines on atopic eczema self-management in primary care in the UK. *BMJ open* 2019a 1;9(7):e025220.
13. Hon KL, Kam WY, Leung TF, et al. Steroid fears in children with eczema. *Acta Paediatrica* 2006;95(11):1451-5.
14. Cowdell F. Knowledge mobilisation: an ethnographic study of the influence of lay mindlines on eczema self-management in primary care in the UK. *BMJ open* 2018;8(8):e021498.
15. Thomas KS, Stuart B, O'Leary CJ, et al. Validation of treatment escalation as a definition of atopic eczema flares. *PloS one* 2015;10(4):e0124770.
16. Smith SD, Hong E, Fearns S, *et al*. Corticosteroid phobia and other confounders in the treatment of childhood atopic dermatitis explored using parent focus groups. *Australas J Dermatol* 2010;51:168–74.
17. Miller WR, Lasiter S, Bartlett Ellis R, *et al*. Chronic disease self-management: a hybrid concept analysis. *Nurs Outlook* 2015;63:154–61.

For peer review only - http://bmjopen.bmj.com/site/about/guidelines.xhtml

18. Taylor SJC, Pinnock H, Epiphaniou E, *et al*. A rapid synthesis of the evidence on interventions supporting self-management for people with long-term conditions: PRISMS – Practical systematic RevIew of Self-Management Support for long-term conditions. *Health Services and Delivery Research* 2014;2:1–580.
19. Department of Health. A Mandate from the Government to NHS England. 2014. https://www.gov.uk/government/publications/nhs-mandate-2014-to-2015 Accessed 17.12.19
20. Lawn S, Schoo A. Supporting self-management of chronic health conditions: common approaches. *Patient Educ Couns* 2010;80:205–11.
21. Kennedy A, Bower P, Reeves D, *et al*. Implementation of self-management support for long term conditions in routine primary care settings: cluster randomised controlled trial. *BMJ* 2013;346:f2882.
22. Ersser SJ, Cowdell FC, Latter SM, *et al*. Self-management experiences in adults with mild-moderate psoriasis: an exploratory study and implications for improved support. *Br J Dermatol* 2010;163:1044–9.
23. Ridd MJ, King AJL, Le Roux E, *et al*. Systematic review of self-management interventions for people with eczema. *Br J Dermatol* 2017;177:719–34.
24. Ersser SJ, Cowdell F, Latter S, *et al*. Psychological and educational interventions for atopic eczema in children. *Cochrane Database Syst Rev* 2014:CD004054.
25. Bass AM, Anderson KL, Feldman SR. Interventions to increase treatment adherence in pediatric atopic dermatitis: a systematic review. *J Clin Med* 2015;4:231–42.
26. Pickett K, Loveman E, Kalita N, *et al*. Educational interventions to improve quality of life in people with chronic inflammatory skin diseases: systematic reviews of clinical effectiveness and cost-effectiveness. *Health Technol Assess* 2015;19:1–176.
27. Ward V. Why, whose, what and how? A framework for knowledge mobilisers. *Evid Policy* 2017;13:477–97.
28. Powell A, Davies HT, Nutley SM. Facing the challenges of research informed knowledge mobilization: 'Practising what we preach'? *Public Adm* 2017:1–17.
29. Jackson CL, Greenhalgh T. Co-creation: a new approach to optimising research impact? *Med J Aust* 2015;203:283–4.
30. Ferlie E, Crilly T, Jashapara A, *et al*. Knowledge mobilisation in healthcare: a critical review of health sector and generic management literature. *Soc Sci Med* 2012;74:1297–304.
31. Marshall M, Pagel C, French C, *et al*. Moving improvement research closer to practice: the researcher-in-Residence model. *BMJ Qual Saf* 2014;23:801–5.
32. Cowdell F. Knowledge mobilisation: an exploratory qualitative interview study to confirm and envision modification of lay and practitioner eczema mindlines to improve consultation experiences and self-management in primary care in the UK. *BMJ open.* 2019b;9(6):e028225.
33. Gabbay J, le May A. Practice-based evidence for healthcare: clinical mindlines. London: Routledge, 2011
34. Polyani M. The tacit dimension. Abingdon: Routledge & Kegan Paul, 1966.
35. Nonaka I, Takeuchi H. The knowledge creating company. New York: Oxford University Press, 1995.
36. Gabbay J, Le May A. Evidence based guidelines or collectively constructed "mindlines?" Ethnographic study of knowledge management in primary care. *Bmj* 2004;329(7473):1013.
37. Wieringa S, Greenhalgh T. 10 years of mindlines: a systematic review and commentary. *Implement Sci* 2015;10:45–55.
38. Flinders M, Wood M, Cunningham M. The politics of co-production: risks, limits and pollution. *Evidence & Policy: A Journal of Research, Debate and Practice* 2016;12(2):261-79.
39. INVOLVE NIHR. Co production. [Available from: http://www.invo.org.uk/current-work/co-production/ Accessed 17.12.19

For peer review only - http://bmjopen.bmj.com/site/about/guidelines.xhtml

40. Co:Create. The Coproduction matrix [Available from: https://www.wearecocreate.com/about Accessed 16.6.20 [Matrix avaialbe on request]
41. Cornwall A. Unpacking 'Participation': models, meanings and practices. *Community Dev J* 2008;43(3):269-83.
42. Palinkas LA, Horwitz SM, Green CA, et al. Purposeful sampling for qualitative data collection and analysis in mixed method implementation research. *Admin Pol Ment Health* 2015;42(5):533-44.
43. Tracy SJ. Qualitative research methods: Collecting evidence, crafting analysis, communicating impact. John Wiley & Sons; 2019.
44. Berger R. Now I see it, now I don't: Researcher's position and reflexivity in qualitative research. *QRJ* 2015;15(2):219-34.
45. Tong A, Sainsbury P, Craig J. Consolidated criteria for reporting qualitative research (COREQ): a 32-item checklist for interviews and focus groups. *Int J Qual Health Care* 2007;19(6):349-357.
46. Dodd M. Motivational Interviewing Ideas for Peer Mentors. https://niatx.net/toolkits/system/IA_MIforPeerMentors.pdf Accessed 17.12.19
47. National Social Marketing Centre What is Social Marketing? https://www.thensmc.com/content/what-social-marketing-1 Accessed 17.12.19
48. Walsh DC, Rudd RE, Moeykens BA, Moloney TW. Social marketing for public health. *Health Aff* 1993;12(2):104-19.
49. Cheng H, Kotler P, Lee N, editors. Social marketing for public health: global trends and success stories. Jones & Bartlett Learning; 2011.
50. Evans WD. How social marketing works in health care. *BMJ* 2006;332(7551):1207-10.
51. Luca NR, Suggs LS. Theory and model use in social marketing health interventions. *J. Health Commun* 2013 Jan;18(1):20-40.
52. Doran N, Fox F, Rodham K, et al. Lost to the NHS: a mixed methods study of why GPs leave practice early in England. *Br J Gen Pract*. 2016;66(643):e128-35.
53. Ball J, Maben J, Griffiths P. Practice nursing: what do we know? British Journal of General Practice . https://bjgp.org/content/bjgp/65/630/10.full.pdf Accessed 17.12.19
54. Gregório J, Cavaco AM, Lapão LV. How to best manage time interaction with patients? Community pharmacist workload and service provision analysis. *RSAP* 2017;13(1):133-47.
55. Institute of Health Visiting 2019 Caseload https://ihv.org.uk/news_tag/caseload/ Accessed 17.12.19
56. Marie Cunningham A, Shirley A. Mindlines in a digital age. *Educ Prim Care* 2015;26(5):293-6.
57. Launer J. Guidelines and mindlines. *Postgrad Med J* 2015;91(1081):663-4.
58. Lau R, Stevenson F, Ong BN, et al. Achieving change in primary care—causes of the evidence to practice gap: systematic reviews of reviews. *Implement Sci* 2015;11(1):40.
59. Gabbay J, le May A. Mindlines: making sense of evidence in practice. BJGP https://bjgp.org/content/66/649/402.short Accessed 17.12.19
60. Levine D, Bleakley A. Maximising medicine through aphorisms. *Med Educ* 2012;46(2):153-62.
61. McColl MA, Aiken A, Smith K, et al. Actionable nuggets: knowledge translation tool for the needs of patients with spinal cord injury. *CFP* 2015;61(5):e240-8.
62. Snider A. Francis Bacon and the authority of aphorism. *Prose Studies* 1988;11(2):60-71.
63. O'Neill N, Mitchell G, Twycross A. The expert patient. Evidence-Based Nursing. 2016 Feb 16:ebnurs-2016. http://dx.doi.org/10.1136/eb-2016-102325 Accessed 17.12.19
64. Shortell SM, Poon BY, Ramsay PP, et al. A multilevel analysis of patient engagement and patient-reported outcomes in primary care practices of accountable care organizations. *J. G. Intern. Med* 2017;32(6):640-7.

For peer review only - http://bmjopen.bmj.com/site/about/guidelines.xhtml

65. Rieckmann P, Centonze D, Elovaara I, et al. Unmet needs, burden of treatment, and patient engagement in multiple sclerosis: A combined perspective from the MS in the 21st Century Steering Group. *Mult Scler Relat Dis* 2018;19:153-60.
66. Killian L, Coletti M. The role of Universal Health Literacy Precautions in minimizing "medspeak" and promoting shared decision making. *AMA J. Ethics* 2017;19(3):296-303.
67. Kashaf M.S. & McGill E. Does Shared Decision Making in Cancer Treatment Improve Quality of Life? A Systematic Literature Review. *Med Decis Making* 2015;35(8):1037-1048.
68. Schuttelaar ML, Vermeulen KM, Drukker N, et al. A randomized controlled trial in children with eczema: nurse practitioner vs. dermatologist. *Br J Dermatol* 2010;162(1):162-70.
69. Cork MJ, Britton J, Butler L, et al. Comparison of parent knowledge, therapy utilization and severity of atopic eczema before and after explanation and demonstration of topical therapies by a specialist dermatology nurse. *Br J Dermatol* 2003;149(3):582-9.
70. Chinn DJ, Poyner T, Sibley G. Randomized controlled trial of a single dermatology nurse consultation in primary care on the quality of life of children with atopic eczema. *Br J Dermatol* 2002;146(3):432-9.
71. Moore E, Williams A, Manias E, Varigos G. Nurse-led clinics reduce severity of childhood atopic eczema: a review of the literature. *Br J Dermatol* 2006;155 (6):1242-8.
72. Tucker R, Stewart D. An exploratory study of the views of community pharmacy staff on the management of patients with undiagnosed skin problems. *Int J Pharm Pract* 2015;23(6):390-8.
73. Carr A, Patel R, Jones M, Suleman A. A pilot study of a community pharmacist intervention to promote the effective use of emollients in childhood eczema. *Pharm J* 2007;278:17.
74. Ward PR, Bissell P, Noyce PR. Medicines counter assistants: roles and responsibilities in the sale of deregulated medicines. *Int J Pharm Pract* 1998;6(4):207-15.
75. Rutter P. Role of community pharmacists in patients' self-care and self-medication. *Integr Pharm Res Prac* 2015;4:57.

For peer review only

BMJ Open Co-creation submitted Dec 2019 (R1 16.6.20 Clean copy)

- Holistic: Coproduction should happen at every stage
- Resourced: Meaningful and effective coproduction deserves and requires sufficient resource
- Transparent: Coproduction should have a clear and transparent remit i.e.: overall aims, limitations, expectations and commitment
- Inclusive: Coproduction should involve a wide range of people (e.g.: practitioners, customers, future users, the wider community), capturing individual and differing views
- Iterative: Coproduction should be reciprocal, repeated, and progressive, always adapting and building upon what came before
- Positive: Coproduction should be mutually beneficial and an overall positive experience
- Equal: Each participant and their contribution should be valued equally
- Sustainable: Meaningful coproduction should have a genuine sustainable impact on the project

Table 1: Eight principles of the Co:Create Coproduction Matrix

For peer review only

For peer review only - http://bmjopen.bmj.com/site/about/guidelines.xhtml

Role	Gender	Age range
Lay person	F	35-44
Lay person	M	Under 16
Lay person	F	Under 16
Lay person	M	Over 55
Lay person	F	Over 55
Lay person	F	25-34
Lay person	F	45-54
Lay person	F	25-34
Lay person	F	45-54
Lay person	F	35-44
Dermatology specialist nurse	F	45-54
General practitioner	F	25-34
General practitioner trainee	M	25-34
Practice nurse	F	45-54
Medical student	F	17-24
Pharmacist	M	25-34
General practitioner trainee	F	25-34
Primary care nurse	F	45-54
Pharmacist	M	45-54
Pharmacy counter assistant	F	35-44
Facilitator	F	45-54
Researcher, facilitator and nurse	F	45-54

Table 2: Demographic details of the co-creation group

For peer review only

Workshop	Attendees	Time	Activity
1	Practitioners, researcher and facilitator	2.5 hours	• Introductions and clarification of purpose of workshop.<br>• Discussion and questions about the circulated article and eczema mindlines illustrations.<br>• Selection from a choice of ordinary postcards that co-creators thought represented a "good" eczema consultation; each person briefly presented their thoughts.<br>• Small group exercise to identify behaviours that patients and practitioners should start, stop and continue to bring about an improvement in eczema consultations and self-management.<br>• Identification of initial priorities for mindline amendment.
2	Lay people, researcher and facilitator	3 hours	• As above
3	Lay people, practitioners, researcher and facilitator	One day	• Introductions and clarification of purpose of workshop<br>• Recap and discussion about the process and outcomes of workshops one and two.<br>• Co-creators individually reviewed priorities for mindline amendment derived from sessions 1 and 2 (Table 4) and ranked these in order of which they considered most likely to lead to improvement in consultation experience and self-management.<br>• Individuals worked in three mixed subgroups to identify their top three priorities.<br>• Whole group reconvened and each subgroup presented their rationale and choice for their top three after which, through consensus activity, a final three priorities for action were agreed.<br>• Subgroups worked with a range of creative resources to contemplate how lay and practitioner eczema mindlines may best be amended.

For peer review only - http://bmjopen.bmj.com/site/about/guidelines.xhtml

Table 3: Details of co-creation workshops

**Priorities for mindline amendment**	**Facets**
Prioritise eczema	Be attentive, make eczema the primary reason for consultation, offer or go for follow-up
Manage eczema as a long-term condition	Educate, explain, avoid quick fixes, don't expect a cure, "get control, keep control"
Prepare for each consultation	Be aware of patient history, offer facts and good explanation, plan what to say, know what you want to achieve
Be consistent with treatment	Be concordant, know products and how to use them, agree realistic regimens, be truthful, don't waste, try over the counter products, understand local formulary
Work together	Listen and question, acknowledge expertise, understand burden of treatment, plan, get control, keep control
Get the right emollient	Be familiar with products, offer choice, agree feasible regimen
Use topical steroids appropriately	Understand risk and benefits, use for flares, use the best product for the optimum time

Table 4: Priorities for and facets of eczema mindline amendment

For peer review only

- Holding 'pop ups' of eczema information in places not currently targeted
- Having simple information available in skin care sections of supermarkets
- Targeting information at 'Health Living Pharmacies'
- Using "Steroid Sam the emoji" in information to illustrate topical steroids in a more positive light
- Making more use of the POEM (Patient Oriented Eczema Measure) / Children's Dermatology Life Quality Index to so parents can be more confident in "proving" impact of eczema during consultations
- Offering a "recipe" for better skin
- Build on existing resources for example Dr Ranj
  https://www.youtube.com/watch?v=lklekfkoSbI
  https://www.youtube.com/watch?v=xM6XaL0gaho

Table 5: Initial ideas for mindline amendment strategies

For peer review only

For peer review only - http://bmjopen.bmj.com/site/about/guidelines.xhtml

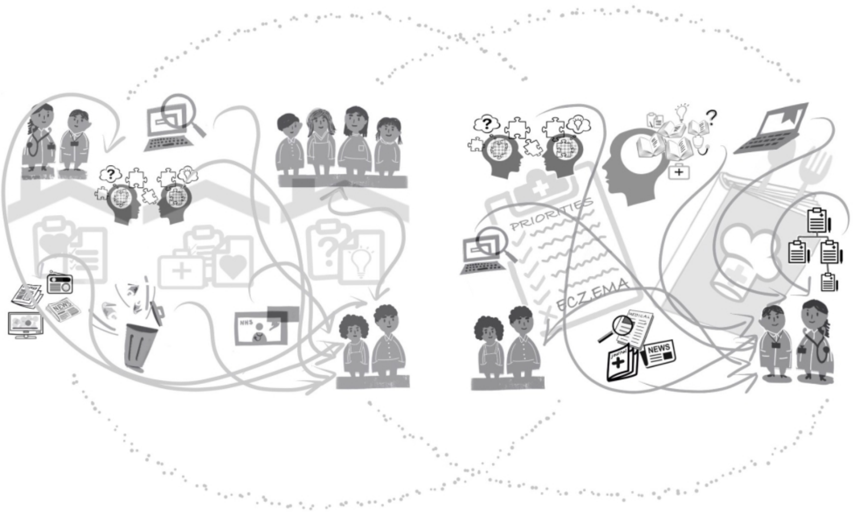

Lay (left side) and practitioner (right side) eczema mindlines and inter-relationship between the two.

394x295mm (132 x 132 DPI)

**Practitioners suggested that lay people should …………..**		
**Start**	**Continue**	**Stop**
Adhering to regular emollient use	Asking questions and challenging the practitioner	Having eczema as a secondary reason for consultation
Be truthful about their concerns	Going to see their practitioner	Stopping regular emollients
Being forceful if not being listened to, asking questions	Asking for advice	Agreeing to care plans they could or would not follow
Committing to the agreed plan of care	Being invested in their skin, wanting to look after it	Waiting for flares before coming for review
Accessing the evidence not the gossip	Be honest with self and practitioner	Not persevering with advice
Realising it's a chronic condition	Listening, writing it down	Using known "triggers"
Finding the right emollient for them	Owning their bodies	Listening to the ill informed
**Practitioners suggested that practitioners should …………..**		
**Start**	**Continue**	**Stop**
Recognising their limitations, accessing education and referring for help	Educating, explaining, supporting and reinforcing messages	Dismissing it as "only" eczema
Explain *how* and *when* to use treatments, monitor side effects and efficacy and give practical tips on useage	Validating concerns	Prescribing products that are impossible to use
Advising about over-the-counter products	Prescribing sufficient emollient prescribing	Viewing all patients as the same
Listening and empathising	Reiterating it's a long-term condition	Thinking eczema is one issue, it effects more than just skin
Signpost or refer for further information or treatment	Reassuring, there will be flares and good times	Under-prioritising how eczema affects patient's lives
Explaining chronicity, encourage regular review	Provide treatment options	Not looking at patient history relevant to consultations
Be familiar with products and provide patients with a selection of emollients so they find the best for them	Myth busting	Presenting cheapest option that doesn't fulfil patient need
Lobbying for products people can use	Recognising impact of eczema on quality of life	Underusing steroids
**Lay people suggested that lay people should …………..**		
**Start**	**Continue**	**Stop**
Preparing for the consultation, what do I want to achieve?	Providing updates	Assuming they know all the answers
Talking to them about long-term care	Choosing the right practitioner for the problem	Expecting a cure
Asking confidently, robustly for what you need and expecting questions to be answered	Trying what you have been asked to try irrespective of first impressions	Holding back, feel confident to say what needs to be said
**Lay people suggested that practitioners should …………..**		
**Start**	**Continue**	**Stop**

For peer review only - http://bmjopen.bmj.com/site/about/guidelines.xhtml

BMJ Open Co-creation submitted Dec 2019

Being more approachable to patients knowledge, requests and understanding	Prescribing what works for the patient	Fobbing us off
Explain *how* to apply emollients and corticosteroids, explain the options	Prescribing as the patient wants	Trying to manage it in primary care if you have reached your limit
Being more realistic about the burden of treatment	Educating themselves clinically	Trivialising and dismissing the condition
Referring patients when prescribed treatments are not sufficient	Being open minded, each patient is unique	Telling us not to scratch
Having a better understanding of medication that's available	Educate themselves about the impact on lives	Treating just our skin, treat the whole person
Knowing the person's eczema history	Continue to educate themselves clinically	Thinking about cost so much
Be equipped with patient history	Value the patient's experience	Making false promises

Supplementary information 1: Practices that lay people and practitioners should start, stop and continue to improve consultation experiences.

For peer review only

Eczema is more than just dry skin

- 1 in 5 babies and children have atopic eczema
- A tendency to atopic eczema often runs in families
- There is no one cause
- Faulty skin barrier means the skin is dry, itchy and inflamed
- Eczema is made worse by ‘triggers’ which can be difficult to avoid

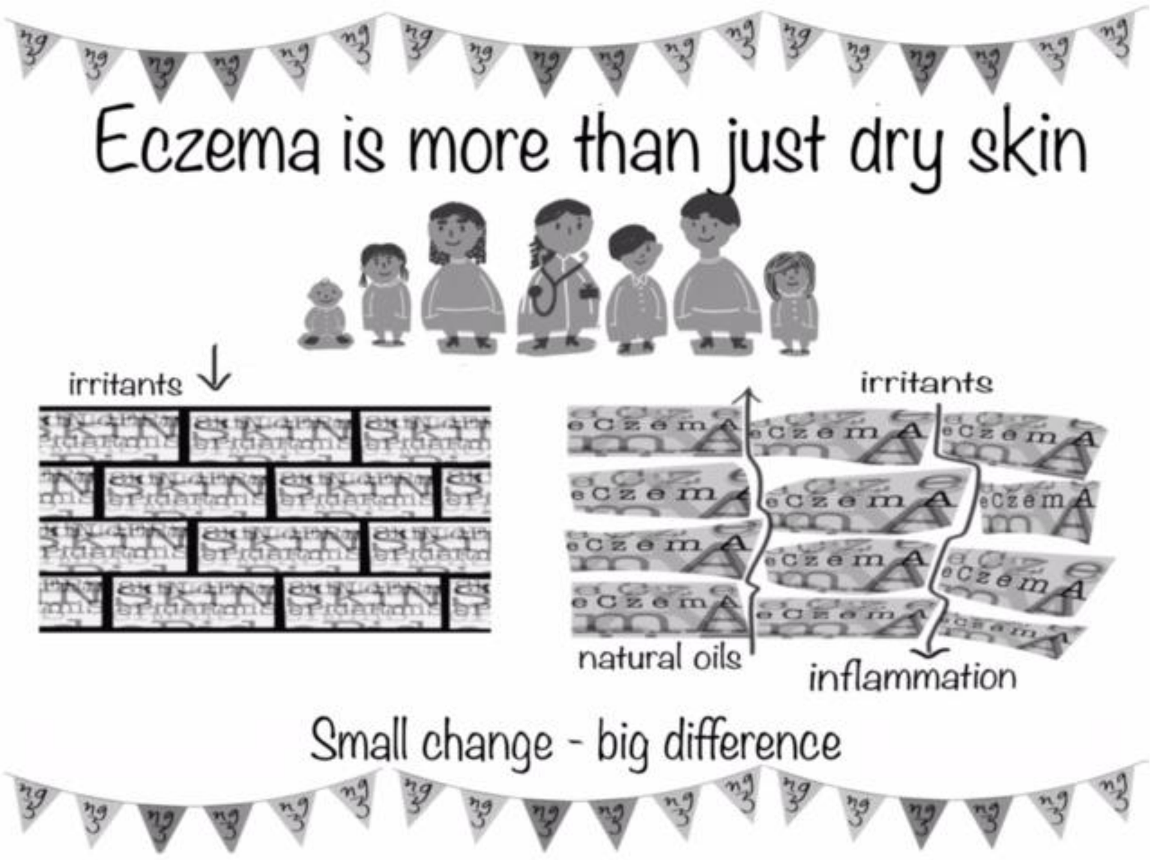

Eczema doesn't just go away

- Children with eczema will always have dry skin
- Dry skin is usually due to skin inflammation
- Skin inflammation makes the skin itchy
- Itchy skin leads to scratching which cause skin damage and more itching
- Constant scratching can result in skin damage, this can be prevented with correct eczema treatment
- Moisturisers should be used every day, even when skin is clear

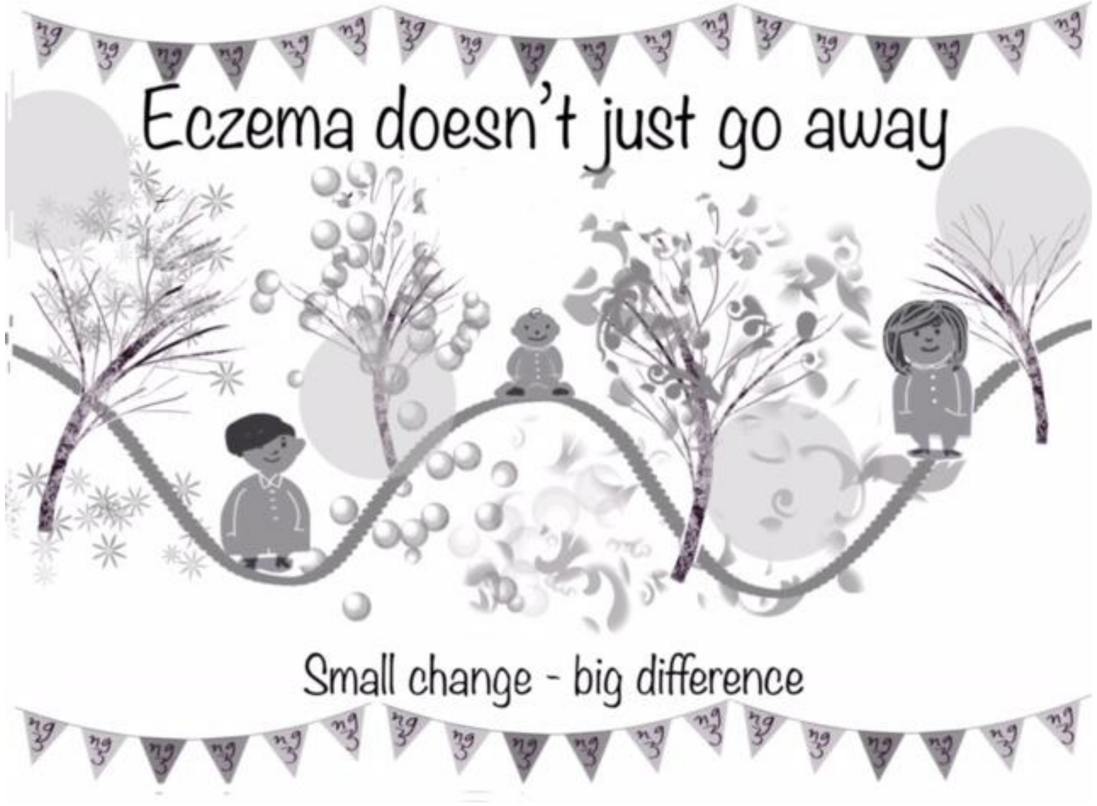

Moisturisers are for everyday

- Moisturisers (emollients) are the first line treatment for dry skin
- Emollients should be used every day, even when skin is clear
- Avoid soaps and bubble baths
- Best emollient is the one that suits your child
- You may need different emollients depending on skin dryness or time of year

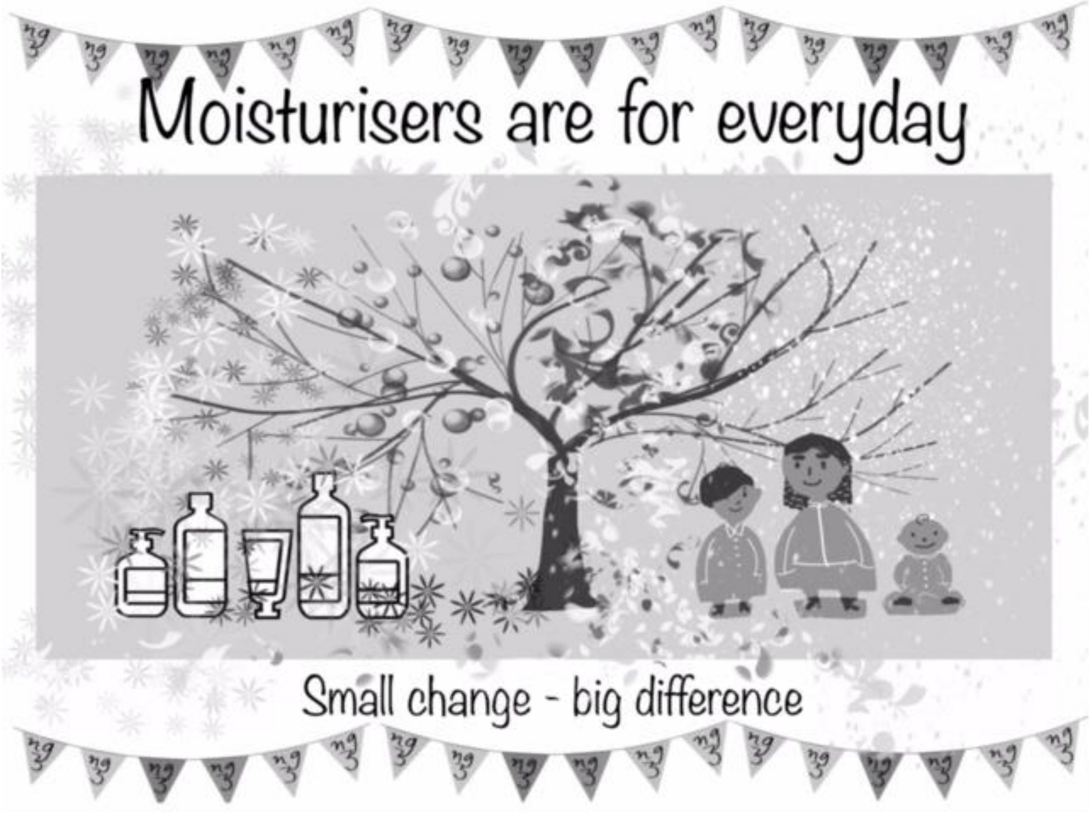

Steroid creams are okay when you need them

- Topical steroids are advised by the NHS as first-line treatments for eczema flares
- They are usually used for short treatment bursts
- It is important to use enough topical steroid, the finger tip unit can be a useful guide, which can be found in the leaflet in the steroid pack
- Topical steroids only need to be applied once a day.
- Topical steroids can have side effects but only if used for long periods and in large quantities in delicate areas such as the face. Your child is more likely to cause damage to their skin due to scratching

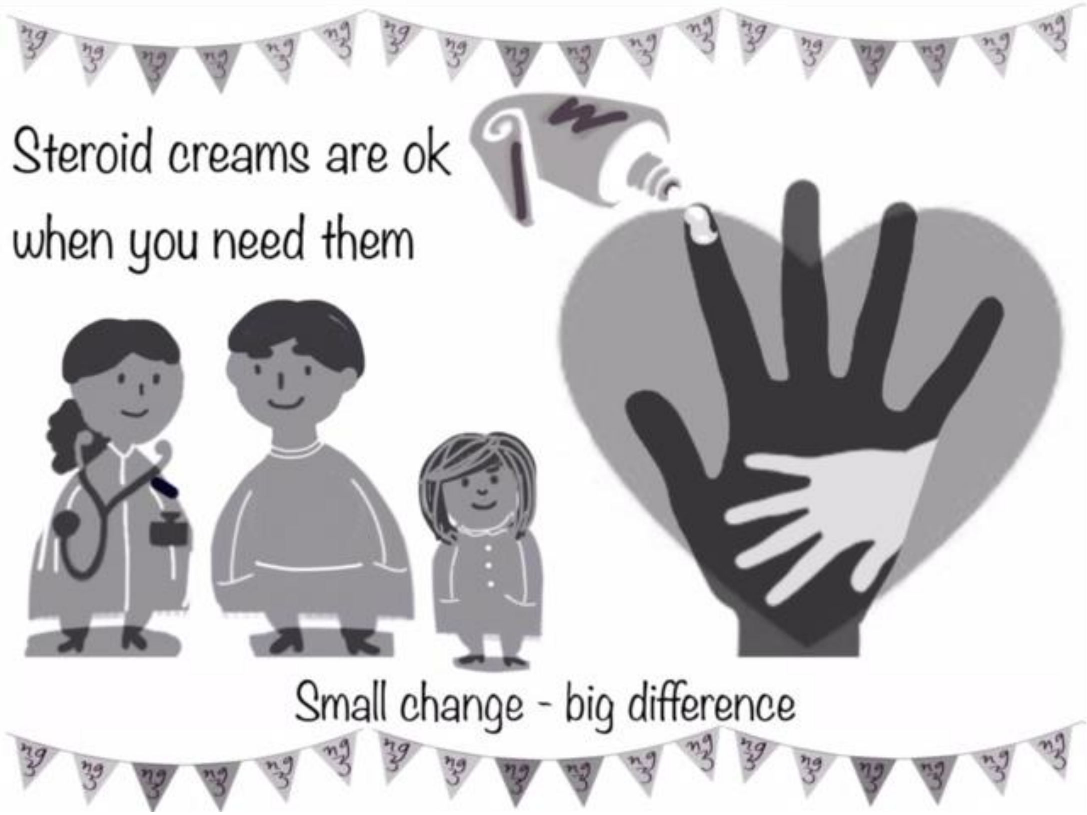

You know your child’s eczema best

- Your pharmacist, health visitor, nurse or GP can help you to manage your child’s eczema
- Book an appointment just to discuss your child’s eczema
- Write down how you currently manage your child’s eczema on a daily basis, use this to discuss how treatment can be improved
- Try to keep a record child’s eczema and how it effects them
- If your child is prescribed topical steroids, ask how much, how often, how to apply and when to stop. Book a review appointment for 2 weeks

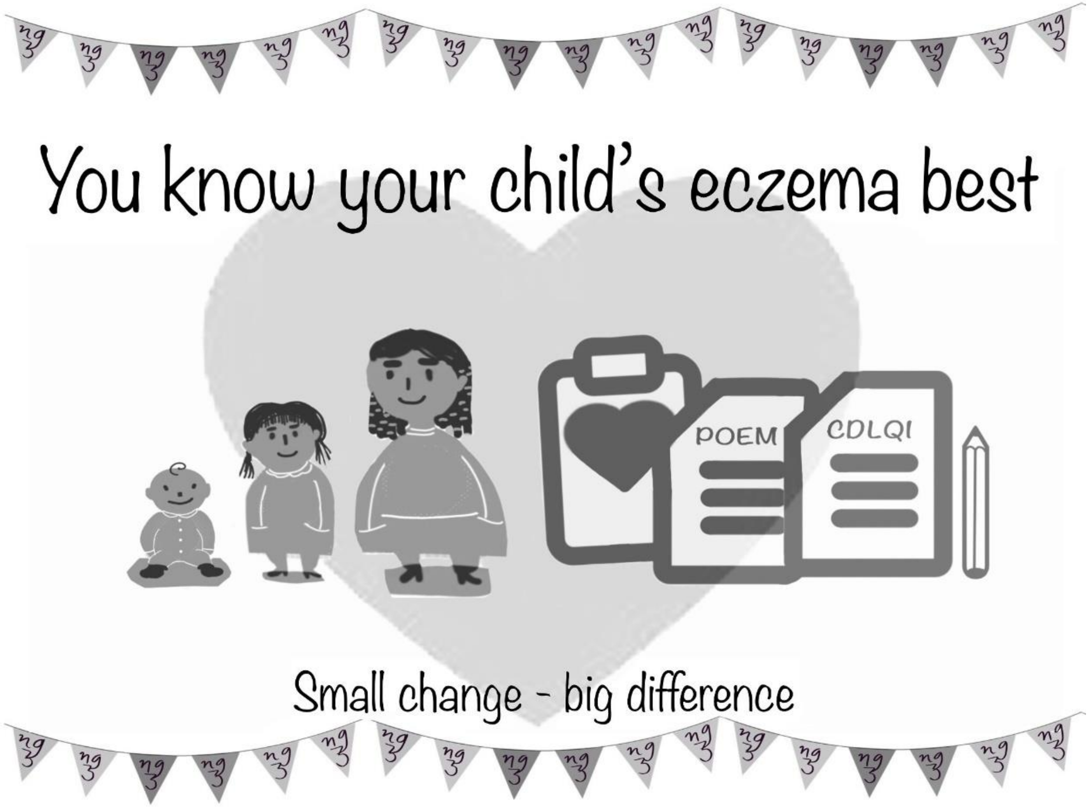

BMJ Open co-creation eczema mindlines COREQ 17.12.19

COREQ Statement

Knowledge mobilisation: An ethnographic study of the influence of practitioner mindlines on eczema self-management in primary care in the United Kingdom

Statement	Page no
**Domain 1: Research team and reflexivity**<br>*Personal Characteristics*<br>1. Interviewer/facilitator Which author/s conducted the interview or focus group?<br>Co-creation workshops were led by FC and CL	6
2. Credentials What were the researcher's credentials? E.g. PhD, MD<br>DProf, RN	Title page
3. Occupation What was their occupation at the time of the study?<br>Professor of Nursing and Health Research	Title page
4. Gender Was the researcher male or female?<br>Female	Title page
5. Experience and training What experience or training did the researcher have?<br>I have extensive experience in qualitative research.	Title page
Relationship with participants<br>6. Relationship established Was a relationship established prior to study commencement?<br>I established rapport with each co-creator through the recruitment process and at the beginning of each group	6 and Table 3
7. Participant knowledge of the interviewer<br>What did the participants know about the researcher? e.g. personal goals, reasons for doing the research<br>I provided an information sheet, explained about myself and the study and answered questions.	6
8. Interviewer characteristics What characteristics were reported about the interviewer/facilitator? e.g. Bias, assumptions, reasons and interests in the research topic<br>Participants were aware, and it is reported in the manuscript, that I am a Registered Nurse with an interest in how eczema knowledge is developed and shared between patients and practitioners in primary care and that this was a publically funded study.	6
**Domain 2: study design**<br>*Theoretical framework*<br>9. Methodological orientation and theory<br>What methodological orientation was stated to underpin the study? e.g. grounded theory, discourse analysis, ethnography, phenomenology, content analysis<br>In the methods section I explain that this is a co-creation study investigating mindline amendment	6
Participant selection<br>10. Sampling How were participants selected? e.g. purposive, convenience, consecutive, snowball<br>Purposive sampling to ensure a mix of co-creators	6
11. Method of approach How were participants approached? e.g. face-to-face, telephone, mail, email<br>HEI website with mass sharing via word of mouth and media posts, professional networks and an existing dermatology patient panel	6
12. Sample size How many participants were in the study?<br>n = 22	6
13. Non-participation How many people refused to participate or dropped out? Reasons?<br>.	NA
Setting<br>14. Setting of data collection Where was the data collected? e.g. home, clinic, workplace<br>Accessible conference centre and via email / telephone	6
15. Presence of non-participants Was anyone else present besides the participants and researchers?	NA
16. Description of sample What are the important characteristics of the sample? e.g. demographic data, date<br>Interview participants were sampled by profession, gender and years in practice.	Table 2
Data collection<br>17. Interview guide Were questions, prompts, guides provided by the authors? Was it pilot tested?	NA

For peer review only

BMJ Open co-creation eczema mindlines COREQ 17.12.19

18. Repeat interviews Were repeat interviews carried out? If yes, how many?	NA
19. Audio/visual recording Did the research use audio or visual recording to collect the data?	No
20. Field notes Were field notes made during and/or after the interview or focus group?<br>Notes and flip charts used	7
21. Duration What was the duration of the interviews or focus group?	½ - 1 day<br>Table 3
22. Data saturation Was data saturation discussed?	NA
23. Transcripts returned Were transcripts returned to participants for comment and/or correction?<br>Data summaries were circulated to group members for comment, supplementation and modification	7
**Domain 3: analysis and findings**<br>*Data analysis*<br>24. Number of data coders How many data coders coded the data?<br>Data analysis was completed by FC and CL with the co-creation group	7
25. Description of the coding tree Did authors provide a description of the coding tree?<br>No	NA
26. Derivation of themes Were themes identified in advance or derived from the data?<br>Findings were derived from the data	7
27. Software What software, if applicable, was used to manage the data?<br>No	NA
28. Participant checking Did participants provide feedback on the findings?<br>Yes	7
Reporting<br>29. Quotations presented Were participant quotations presented to illustrate the themes / findings? Was each quotation identified? e.g. participant number<br>Quotations are provided	8-15
30. Data and findings consistent Was there consistency between the data presented and the findings?<br>Yes.	8-15
31. Clarity of major themes Were major themes clearly presented in the findings?<br>Yes.	8-15
32. Clarity of minor themes Is there a description of diverse cases or discussion of minor themes?<br>Yes,	8-15

For peer review only
