# Supplementary material for: Knowledge mobilisation: a UK co-creation study to devise strategies to amend lay and practitioner atopic eczema mindlines to improve consultation experiences and self-management practices in primary care
Source: BMJ Open. 2020 Sep 28;10(9):e036520. doi: 10.1136/bmjopen-2019-036520 (PMC7523205; doi:10.1136/bmjopen-2019-036520)
Supplement: Reviewer comments [file bmjopen-2019-036520.reviewer_comments.pdf]

PEER REVIEW HISTORY

BMJ Open publishes all reviews undertaken for accepted manuscripts. Reviewers are asked to complete a checklist review form (**http://bmjopen.bmj.com/site/about/resources/checklist.pdf**) and are provided with free text boxes to elaborate on their assessment. These free text comments are reproduced below.

ARTICLE DETAILS

**TITLE (PROVISIONAL)**	Knowledge mobilisation: a UK co-creation study to devise strategies to amend lay and practitioner atopic eczema mindlines to improve consultation experiences and self-management practices in primary care
**AUTHORS**	Cowdell, Fiona; Ahmed, Taheeya; Layfield, Carron

VERSION 1 – REVIEW

**REVIEWER**	Regina Fölster-Holst<br>Dermatology<br>University clinics of Schleswig-Holstein<br>24 105 Kiel
**REVIEW RETURNED**	05-Apr-2020

**GENERAL COMMENTS**	This is really an interesting investigation, which shows once again that the consultation does not always lead to patient satisfaction.<br>The article should be published.<br>Questions / comments<br>1. Why weren't there dermatologists at this workshop?<br>2. The work of education/training on atopic eczema should also be mentioned in the discussion, because ultimately, the topics are also addressed in these meetings.

**REVIEWER**	Fiona Collier<br>Dr.Fiona Collier<br>Locum GP and GPSI Dermatology<br>NHS Forth Valley<br>Scotland
**REVIEW RETURNED**	23-Apr-2020

**GENERAL COMMENTS**	This co-creation study on lay and medical eczema mindlines is clearly and engagingly written. It displays rigour and credibility in its design, and also provides a clear explanation of the relatively new methodology of co-creation on which it is based. There is a logical progression from data to the findings and conclusions. The process of trying to elicit and align practitioner and lay/patient mindlines is described in sufficient detail that it would be straightforward to replicate it for other chronic conditions, and this aspect makes the paper important outwith a Dermatology-orientated readership.A particular strength is the commitment to putting this newly-constructed knowledge into practice. The resources at the end of the paper, posters expanding on the 5 key messages, were very clear and easily understood by lay audiences of all reading ages, and would be helpfu consultation aids for practitioners working with people with eczema.<br>There were a couple of small points: 1. I didn't find Figure 1

	'Lay/Practitioner Mindlines to be clear in its intent or meaning, and I don't think it adds much. 2.I think it would be useful, from the point of view of reflexivity, for FC to mention she has previous publications in the field of Eczema Mindlines.

VERSION 1 – AUTHOR RESPONSE

Reviewer: 1<br>Reviewer Name: Regina Fölster-Holst	
This is really an interesting investigation, which shows once again that the consultation does not always lead to patient satisfaction. The article should be published.	Thank you for these positive comments
1. Why weren't there dermatologists at this workshop?	This is an interesting point. I have worked closely with consultant dermatologists at other stages of this study. The focus here is on getting the fundamental aspects of eczema treatment right in primary care settings and for this reason it was more appropriate to engage with primary care staff.
2. The work of education/training on atopic eczema should also be mentioned in the discussion, because ultimately, the topics are also addressed in these meetings.	I agree, training and education are also important, for both patients and practitioners and have added a sentence about this to the discussion.
Reviewer: 2<br>Reviewer Name: Fiona Collier	
This co-creation study on lay and medical eczema mindlines is clearly and engagingly written. It displays rigour and credibility in its design, and also provides a clear explanation of the relatively new methodology of co-creation on which it is based. There is a logical progression from data to the findings and conclusions. The process of trying to elicit and align practitioner and lay/patient mindlines is described in sufficient detail that it would be straightforward to replicate it for other chronic conditions, and this aspect makes the paper important outwith a Dermatology-orientated readership. A particular strength is the commitment to putting this newly-constructed knowledge into practice. The resources at the end of the paper, posters expanding on the 5 key messages, were very clear and easily understood by lay audiences of all reading ages, and would be helpful consultation aids for practitioners working with people with eczema.	Thank you, we really appreciate your comments. In particular your views as a GP are so important to us. This is an ongoing study which is intended to influence care on many levels.
1. I didn't find Figure 1 'Lay/Practitioner Mindlines to be clear in its intent or meaning, and I don't think it adds much.	We have added a little more explanation of the illustration
2. I think it would be useful, from the point of view of reflexivity, for FC to mention she has previous publications in the field of Eczema Mindlines.	Thank you, I've added this in the introduction

VERSION 2 – REVIEW

**REVIEWER**	Regina Fölster-Holst<br>University clinics of Schleswig-Holstein<br>Campus Kiel<br>Germany
**REVIEW RETURNED**	29-Jun-2020

**GENERAL COMMENTS**	In my first review I already mentioned that a dermatologist would improve the study. I would like the authors to mention this in the discussion.

**REVIEWER**	Fiona Collier<br>NHS Forth Valley<br>Scotland
**REVIEW RETURNED**	20-Jun-2020

**GENERAL COMMENTS**	Well-written paper decribing innovative study with high relevance to everyday medical practice.
